# Inhibition of cannabinoid receptor type 1 sensitizes triple-negative breast cancer cells to ferroptosis via regulating fatty acid metabolism

**DOI:** 10.1038/s41419-022-05242-5

**Published:** 2022-09-21

**Authors:** Pengyun Li, Qiaohong Lin, Shiyang Sun, Ning Yang, Yu Xia, Shengjie Cao, Wenjuan Zhang, Qian Li, Haoxin Guo, Maoxiang Zhu, Yilong Wang, Zhibing Zheng, Song Li

**Affiliations:** 1 Laboratory of Computer-Aided Drug Design & Discovery, Beijing Institute of Pharmacology and Toxicology, Beijing, 100850 China; 2 Department of Chemistry, MOE Key Laboratory of Bioorganic Phosphorus Chemistry & Chemical Biology, Tsinghua University, Beijing, 100084 China; 3 Beijing Key Laboratory for Radiobiology, Beijing Institute of Radiation Medicine, Beijing, 100850 China

**Keywords:** Phenotypic screening, Breast cancer, Cell death

## Abstract

Triple-negative breast cancer (TNBC) is a heterogeneous subtype of breast cancer that displays highly aggressive with poor prognosis. Owing to the limited targets and drugs for TNBC clinical therapy, it is necessary to investigate the factors regulating cancer progression and develop novel therapies for cancer treatment. Ferroptosis, a nonapoptotic form of programmed cell death characterized by accumulation of iron-dependent peroxidation of phospholipids, is regulated by cellular metabolism, redox homeostasis, and various cancer-related signaling pathways. Recently, considerable progress has been made in demonstrating the critical role of lipid metabolism in regulating ferroptosis, indicating potential combinational therapeutic strategies for cancer treatment. In this study, by drug combination screen of lipid metabolism compounds with ferroptosis inducers in decreasing TNBC cell viability, we found potent synergy of the CB1 antagonist rimonabant with erastin/(1 S, 3 R)-RSL3 (RSL3) in inhibiting TNBC cell growth both in vitro and in vivo via promoting the levels of lipid peroxides, malondialdehyde (MDA), 4-hydroxynonenal (4-HNE) and cytosolic reactive oxygen species (ROS) production, enhancing intracellular glutathione (GSH) depletion and inducing G1 cell cycle arrest. We identified that inhibition of CB1 promoted the effect of erastin/RSL3 on inducing ferroptosis and enhanced their inhibitory effect on tumor growth. Using RNA-Seq, fatty acid analyses and functional assays, we found that CB1 regulated stearoyl-CoA desaturase 1 (SCD1)- and fatty acyl desaturase 2 (FADS2)-dependent fatty acid metabolism via phosphatidylinositol 3 kinase (PI3K)-AKT and mitogen-activated protein kinase (MAPK) signaling pathways to modulate ferroptosis sensitivity in TNBC cells. These data demonstrate that dual targeting of CB1 and ferroptosis could be a promising therapeutic strategy for TNBC.

## Introduction

Breast cancer is one of the most commonly diagnosed malignancies and one of the leading causes of cancer-related deaths in women. Triple-negative breast cancer (TNBC) accounts for ~15–18% of all breast cancers and is characterized with strong invasiveness, rapid disease progression, recurrence after treatment and poor clinical prognosis [1]. Treatment of TNBC remains clinically challenging, which usually involves chemotherapy, such as taxanes and anthracyclines [2]. Unfortunately, a majority of patients with TNBC develop acquired chemoresistance after preoperative chemotherapy, resulting in relapse with rapid disease progression and a high mortality rate within 3–5 years [3]. Recent preclinical and clinical studies demonstrate the efficacy of Poly ADP-ribose polymerase (PARP) inhibitors such as Olaparib, Talazoparib, Niraparib as monotherapy for patients with breast cancer susceptibility gene 1/2 (BRCA1/2)-mutated ovarian and breast cancers including TNBC [2]. Only 10–15% of TNBC patients, however, harbor BRCA1/2 mutations, while the remainders have different mutations or copy number alterations in different oncogenes or tumor suppressor genes [4]. Therefore, there is an urgent need to explore novel TNBC therapeutic treatment.

Ferroptosis, a nonapoptotic form of programmed cell death, is characterized by accumulation of iron-dependent peroxidation of phospholipids [5]. Emerging evidence have manifested the pivotal role of ferroptosis in suppressing breast tumor growth by modulating various tumor properties [6, 7]. It has been reported that TNBC cells display a unique metabolic state of iron and GSH homeostasis, which can enhance their sensitivity to ferroptosis [8]. Long-chain acyl-CoA synthetase 4 (ACSL4), a critical factor which links arachidonic acid (AA) and adrenic acid (AdA) to phosphatidylethanolamine (PE) was shown to be highly expressed in basal-type breast cancer cells and regulated ferroptosis sensitivity [9]. xCT, a functional subunit of the cystine/glutamate antiport (system Xc-), Hasegawa et al. found that the inhibition of mucin 1 (MUC1)/xCT signaling pathway can induce GSH level imbalance and ROS-mediated ferroptosis in TNBC cells, resulting in killing tumor cells and reducing their self-renewal ability [10]. At the same time, with the elaboration of various ferroptosis inducers, developing ferroptosis inducing agents or drugs to inhibit breast cancer cells is also the focus of current research. Ma et al. found that synergy of siramesine and lapatinib can induce the death of breast cancer cells characterized by lipid peroxides production and increased FeCl_3_, which can be reversed by ferrostatin-1 and deferoxamine (DFO) [11]. The latest research reported that the combinational treatment of the bromodomain and extraterminal (BET) inhibitor JQ1 and the proteasome inhibitor BTZ specifically induces TNBC ferroptosis and significantly inhibits the growth of TNBC tumors [8]. These data indicate that targeting ferroptosis might be a promising therapeutic strategy for TNBC.

Increasing studies have shown that dysregulation of lipid metabolism contributes to cancer progression and cancer drug resistance [12–14]. As ferroptosis is characterized with the accumulation of lethal lipid peroxides through the peroxidation of phospholipids containing polyunsaturated fatty acids (PUFA), recent studies have classified that reprograming of lipid metabolism is closely related to the ferroptosis sensitivity [15, 16]. Multiple genes and compounds related to lipid metabolism can induce or inhibit ferroptosis in cancer cells. Intriguingly, lipid metabolic enzymes are inherently druggable which offering a wealth of new targets, and various pharmacological inhibitors have been developed and utilized in cancer clinical practice [17], motivating us for further exploration of combining the ferroptosis induction with the metabolic inhibition for cancer therapies. In the past few decades, the endocannabinoid system has attracted widespread interest in multiple diseases applications which consists of cannabinoid receptors (CB) receptors, endocannabinoids, related enzymes and endogenous cannabinoids bind to CB1 and CB2, thus regulating various metabolic pathways [18]. Importantly, hyperactivation of CB1 has been identified in various cancer types and CB1 has been extensively reported correlated with cancer development and its clinical outcome [19]. Moreover, accumulating studies have shown that natural and synthetic (endo)cannabinoids exert anticancer effect by decreasing proliferation, inducing cell cycle arrest and apoptosis, inhibiting neovascularization, invasion, and abrogating chemoresistance involved in regulating downstream signaling such as PI3K-AKT and MAPK signaling pathways [20, 21], suggesting that targeting CB1 receptors might be a promising anticancer strategy. Although it is widely established that CB1 has diverse effects contributing to tumor progression, its effect on ferroptosis has not ever been explored.

In this study, we performed drug combination screening of lipid metabolism compounds with erastin/RSL3 on decreasing TNBC cells viability to identify potent synergistic combinations. We found that combination of CB1 antagonist rimonabant with ferroptosis inducers exhibited potent synergistic effects. We further investigated the biological function and underlying mechanism, which will help to translate this knowledge into the clinical setting to develop new therapies for TNBC treatment.

## Material and methods

### Chemical and drugs

The small-molecule inhibitors were all purchased from Targetmol, Wellesley Hills, MA, USA including Lipid metabolism compounds library (L2510), CB1 antagonists (AM251, T1915; AM281, T2264; JD5037, T4453; Otenabant, T3530; TM3837, T8511; CB1-IN-1, T5996), ferroptosis inducers (erastin, T1765; RSL3, T3646; ML-210, T8375; FIN56, T4066; CIL56, T4309), PI3K inhibitor (LY294002, T2008) and MEK inhibitor (PD98059, T2623).

### Cell lines

All cell lines used in this study were purchased from the American Type Culture Collection (ATCC, Manassas, VA, USA). All cell lines were authenticated according to the short tandem repeat (STR) profile. The TNBC cell lines (MDA-MB-231, MDA-MB-436, HCC38, Hs578T), luminal cell lines (MCF-7, ZR75-1, T47-D), HER2-enriched cell line SKBR3, and human embryonic kidney (HEK) 293 T cells were grown in Dulbecco’s modified Eagle medium (DMEM; Hyclone, Logan, UT, USA) containing 25 mmol/L glucose (Invitrogen, Carlsbad, CA, USA), while TNBC cell line HCC1937 and luminal cell line BT474 cells were grown in Roswell Park Memorial Institute (RPMI) (Hyclone, USA). TNBC cell line BT549 cells were grown in RPMI (Hyclone, USA) containing insulin (10 μg/mL; Sigma-Aldrich, St. Louis, MO, USA). TNBC cell line BT-20 cells were grown in Eagle’s minimum essential medium (MEM) (Gibco, Grand Island, NY, USA). All cell lines were cultured in medium containing 10% fetal bovine serum (FBS) (Hyclone, USA), 1% penicillin (Invitrogen, USA), and 1% streptomycin (Invitrogen, USA). Human Mammary Epithelial Cells (HMEC) cells were grown in Medium 171 (Invitrogen, USA) containing 5% mammary epithelial growth supplement (MEGS) (Invitrogen, USA). All these cells were cultured at 37 °C and 5% CO_2_.

### Antibodies

The antibodies were purchased from different sources. Rabbit anti-β-actin (20536-1-AP), rabbit anti-cyclin D1 (26939-1-AP), rabbit anti-p21 (10355-1-AP), rabbit anti-Cyclin B1 (55004-1-AP), mouse anti-ERK (67170-1-Ig), rabbit anti-AKT (10176-2-AP) and rabbit anti-Ki67 (27309-1-AP) were purchased from Proteintech, Rosemont, IL, USA; Rabbit anti-CB1 (93815), rabbit anti-SCD1 (2794), and rabbit anti-phos-AKT (T308) (13038) were purchased from Cell Signaling Technology, Boston, MA, USA; Mouse anti-phos-ERK (T202/Y204) (sc-136521) was purchased from Santa Cruz Biotechnology, Dallas, Texas, USA. Rabbit anti-FADS2 (PA5-48353) was purchased from Invitrogen, USA. anti-4-HNE (ab46545) purchased from Abcam, Boston, MA, USA.

### Plasmids and shRNA lentivirus infection

MDA-MB-231 and HCC1937 cells lines that stably overexpressing CB1 were established by lentiviral transduction using pCDH plasmid (System Biosciences, Palo Alto, CA, USA) with the following primers: 5'-CGGGATCCATGAAGTCGATCCTAGATGGCC-3' (forward) and 5'-CGGAATTCTCACAGAGCCTCGGCAGACGTG-3' (reverse). Lentiviral constructs of human CB1 shRNAs, SCD1 shRNAs and FADS2 shRNAs were purchased from Genechem, Shanghai, China. Lipofectamine 3000 reagent was used for transfection of plasmids according to the manufacturer’s instructions (Invitrogen, USA). For shRNA lentivirus infection, lentivirus was generated by transfection of the 293 T producer cell line with the lentiviral vector and packing vector mix (System Biosciences, USA). Lentivirus was collected 48 h later, which was used to infect MDA-MB-231 and HCC1937 cells. Stable cell lines were selected with puromycin (3 μg/mL) after 48 h infection. Pooled clones were screened by western blotting (WB) with anti-CB1, anti-SCD1 and anti-FADS2, respectively. For plasmid transfection, cells were seeded to 70–90% confluent at the time of transfection. Plasmids and P3000 reagent were diluted in Opti-MEM (Invitrogen, USA). The diluted plasmids were mixed with the diluted Lipofectamine 3000. The mixtures were incubated for 15 min at room temperature (25–30 °C) and the mixture was added to cells in each dish. The transfected cells were collected after 24–48 h.

### Cell viability and colony formation assays

For cell viability assay, breast cancer cells were seeded in 96-well plates (100 μL per well) at 5000 cells/well to adhere overnight and then treated with the described drug doses for 72 h, and dimethyl sulfoxide (DMSO) was used as control. Anchorage-dependent cells viability was evaluated by the cell counting kit-8 (CCK-8 Kit) according to the manufacturer’s instructions (Dojindo Laboratories, mamoto Ken, Japan). For colony formation assay, stably transfected MDA-MB-231 and HCC1937 cells or parental MDA-MB-231 and HCC1937 cancer cells were plated in six dishes in triplicate at 2000 cells per well to adhere overnight and then treated with the described drug doses for 10–14 days. During the period of drug treatment, cell medium was replaced every 3 days containing the described drug. Subsequently, colonies were fixed with 4% paraformaldehyde for 25 min, stained with 0.2% crystal violet solution for 30 min at room temperature (25–30 °C). The cells were then washed, dried, and scanned using an HP Scanjet.

### Synergy Index (SI) analysis, drug screening, and drug synergy assays

These 303 compounds predominantly target receptors or enzymes involved in regulating lipid metabolism, including CB1, acetyl-CoA carboxylase (ACC), AMP-activated protein kinase (AMPK), fatty acid amide hydrolase (FAAH), and HMG-CoA reductase (HMGCR), sterol-regulatory element binding proteins (SREBPs), peroxisome proliferators-activated receptor (PPAR), etc. To identify potential candidate synergy pairs, Synergy Index was used to estimate the strength of each drug combination. Synergy Index_(erastin)_ = [Fa_Comb(Cpd. + erastin)_ − Fa_Cpd._]/Fa_erastin_; and Synergy Index_(RSL3)_ = [Fa_Comb(Cpd. + RSL3)_ − Fa_Cpd._]/Fa_RSL3_. The fraction of cells inhibited by the drugs was described as fraction affected (Fa). Fa_Cpd._, Fa_erastin_, Fa_RSL3_, Fa_Comb(Cpd. + erastin)_ and Fa_Comb(Cpd.+RSL3)_ respectively represent the inhibition rate of lipid metabolism compounds, erastin, RSL3, synergy of lipid metabolism compounds with erastin and synergy of lipid metabolism compounds with RSL3 on cell viability compared with vehicle-treated cells. The inhibitory effect of single agents or each drug combination on cell viability is provided in Supplementary Table 1, 2. The Synergy Index_(erastin)_ and Synergy Index_(RSL3)_ for each drug pair are provided in Supplementary Table 3 to reflect the strength of combinational effect in the screen. SI < 1 indicates antagonism; SI > 1 indicates synergism. The size of SI value indicates the effect of drug combination.

The drug screening was performed in 96-well plates, and cell viability was validated with CCK-8 kit after single and combinational treatment of 303 different lipid metabolism compounds with erastin or RSL3 for 72 h according to the manufacturer’s instructions (Dojindo Laboratories, Japan). Potent combinations were defined as those drug pairs induced at least 50% death in TNBC cell lines, and the lipid metabolism compound corresponding Synergy Index_erastin_ > 1 and Synergy Index_RSL3_ > 1.

For drug synergy assays, synergy effect of drug pairs was quantitative defined by the Chou-Talalay equation. Combinational Index (CI) < 1 indicates synergism; CI > 1 indicates antagonism [22]. MDA-MB-231 and HCC1937 cells were treated with different concentrations of two single drugs and combinational treatment for 72 h, respectively, and cell viability was validated with CCK-8 kit according to the manufacturer’s instructions (Dojindo Laboratories, Japan).

### Drug combinations of CB1 antagonists with ferroptosis inducers, and cell death rescue experiments

MDA-MB-231 and HCC1937 cells were seed in 96-well plates for each cell line (TNBC and non-TNBC, ~13 cell lines) at 4000 cells per well, and cell viability assay was conducted with CCK-8 kit after combinational treatment of CB1 antagonists with ferroptosis inducers (erastin, RSL3, ML210, FIN56, CIL56) for 72 h according to the manufacturer’s instructions (Dojindo Laboratories, Japan). For cell death rescue experiments, the effects of various cell death pathway inhibitors were calibrated for each cell line for doses and effects. Ferroptosis inhibitors (Ferrostatin-1, GSH, and Deferoxamine), and cell death inhibitors were used to inhibit apoptosis (zVAD-FMK), necrosis (Necrostatin-1), autophagy (3-Methyladenine) at indicated concentrations 1 h before the treatment with combination therapy.

### Cell cycle assay

MDA-MB-231 and HCC1937 cancer cells (1 × 10^6^ cells) were cultured in six-well dishes to adhere overnight and then treated with rimonabant, erastin, RSL3 as single treatment or their combination (rimonabant + erastin and rimonabant + RSL3) for 48 h. For cell cycle analysis, cells were fixed in 75% ethanol for >18 h at 4 °C, washed with phosphate buffered saline (PBS), and incubated with RNase A (0.2 mg/mL) in PBS. Propidium Iodide was then added to the cell suspension. Samples were analyzed by a FACS calibur Flow Cytometer (Becton Dickinson, Franklin Lakes, NJ, USA).

### Lipid peroxidation measurement

C11-BODIPY dye (10 μM) was used for lipid ROS staining according to the manufacturer’s instructions (D3861; Thermo Fisher Scientific, Waltham, MA, USA) with the positive control, cumene hydroperoxide. In short, stably transfected, or parental MDA-MB-231 and HCC1937 cancer cells were collected after treated with rimonabant, erastin, RSL3 as single treatment or their combination (rimonabant + erastin and rimonabant + RSL3) for 2 days, and 10 μM C11-BODIPY-containing medium for 1 h and the lipid ROS level was determined by flow cytometry analysis (FACS Canto II; BD Biosciences).

### GSH measurements

The level of GSH was measured according to Intracellular GSH Detection Assay Kit (Abcam, USA). MDA-MB-231 and HCC1937 cells (1 × 10^6^) were treated with rimonabant, erastin, RSL3 as single treatment or their combination (rimonabant + erastin and rimonabant + RSL3) and then lysed in GSH assay buffer, and GSH levels were measured according to the manufacturer’s instructions. Colorimetric signals were measured by absorbance at 405 nm using a microplate reader (Molecular Devices, Sunnyvale, CA, USA). The GSH concentration was calculated using a standard curve. Protein quantification was performed using the Pierce BCA Protein Assay Kit (Thermo Fisher Scientific, USA) as described in the manufacturer’s guidelines. The values were then normalized to total protein quantity per lysate sample. Results are expressed as means ± SD of fold changes relative to untreated controls.

### ROS measurements

MDA-MB-231 and HCC1937 cells (1 × 10^6^ cells) were cultured in six-well dishes to adhere overnight and then treated with compound for 48 h. Subsequently, the cell culture medium was removed and 2 mL of diluted 25 μM carboxy-H_2_DCFDA (88-5930-74, Invitrogen, USA) was added to the six-well plate, followed by incubation at 37 °C for 30 min. Cells were gently washed three times with warm PBS and Tert-butyl hydroperoxide (TBHP) was added to the positive control well. ROS levels were determined by flow cytometry analysis using 488 nm excitation wavelength and 525 nm emission wavelength (FACS Canto II; BD Biosciences).

### Evaluation of malondialdehyde (MDA) and 4-hydroxynonenal (4-HNE) level

The relative MDA concentration in cell or tumor lysates was assessed using a Lipid Peroxidation (MDA) Assay Kit (ab118970, Abcam), according to the manufacturer’s instructions. This assay measures MDA reaction with thiobarbituric acid (TBA) that generate a MDA-TBA adduct in a sample. The MDA-TBA adduct can be quantified colorimetrically (OD = 532 nm). Lipid Peroxidation (4-HNE) Assay Kit (Abcam, ab238538) were used to evaluate the concentration of 4-HNE according to the manufacturer’s protocol.

### Generation of drug-tolerant cells

For generation of the MDA-MB-231 and HCC1937 erastin-/RSL3-resistance cell lines, parental cells were cultured in 6 cm dishes to adhere overnight and then treated with erastin (10 μM) and RSL3 (1 μM) for 72 h. The surviving attached cells were trypsinized, washed once in PBS and re-cultured in drug-contained medium. Drug concentration was increased by 25% after treated every 2 weeks until the half maximal inhibitory concentration (IC_50_) of drug-tolerant cells is approximately ten times larger than parental cells.

### RNA isolation and Quantitative real-time-PCR

Total RNA was isolated and extracted using TRIzol reagent according to the manufacturer’s instructions (Invitrogen, USA). MDA-MB-231 and HCC1937 cancer cells were pelleted and washed with 1 × PBS. TRIzol reagent (1 mL) was added for each sample and the lysate was pipetted up and down to homogenize mixture followed by incubation at room temperature (25–30 °C) for 5 min. Chloroform (200 μL) was added and then the mixture was incubated at room temperature (25–30 °C) for 2–3 min. The sample was centrifuged at 12,000 × *g* at 4 °C for 15 min, afterwards transferred 400 μL colorless upper aqueous phase into a new EP tube. Isopropanol (400 μL) was added for each sample incubated at room temperature (25–30 °C) for 10 min. The sample was centrifuged at 12,000 × g at 4 °C for 10 min. The supernatant was slowly poured out and wash once with 1 mL of 75% ethanol prepared with diethyl pyrocarbonate (DEPC) water. The supernatant was discarded and dried, and 40 μL of DEPC water was added to dissolve the RNA and stored at −70 °C. A minimum of 2 μg of total RNA was reverse transcribed into first strand cDNA with oligo (dT) primers using Moloney murine leukemia virus reverse transcriptase (Promega, Madison, WI USA). qPCR was performed in triplicates in a 20 μL reaction mixture containing 10 μL of SYBR Premix Ex Taq Master Mix (2×) (Takara, Shiga-ken, Japan), 0.5 mM of each of the primers and 10 ng cDNA. The relative expression level of the target was calculated using the comparative Ct method. β-actin was used as an internal control to normalize sample differences.

### Animal models for tumor growth

Animal studies were approved by the Institutional Animal Care Committee of Beijing Institute of Biotechnology. Nude mice were purchased from Vital River Laboratory Animal Technology (Beijing, China) and housed in a specific pathogen free (SPF) animal facility. For tumor xenografts, 5 × 10^6^ MDA-MB-231, HCC1937 cells or MDA-MB-231 cell stably transfected with shNC or shCB1 in 0.1 mL PBS were injected subcutaneously into the dorsal flank of 6-week-old female nude mice and the mice were treated consecutively. Tumor size was measured at the indicated time using calipers. Tumor volume was estimated according to the following formula: volume = (longest diameter × shortest diameter^2^)/2. When tumors reached a volume of ~100 mm^3^, the mice were randomly divided into groups and intraperitoneally injected with drugs every 2 days, with an equivalent volume of saline injected in control animals. The mice were weighed, and tumor volumes were measured every 4 days. Tumor growth inhibition (TGI) was utilized to determine the inhibitory strength of drugs treatment on tumor growth. TGI (%) = (*V*c − *V*t)/(*V*c − *V*_0_) * 100, where *V*c and *V*t are the median volume of control and treated groups at the end of the study, respectively, and *V*_0_ is median volume of control at the start of the study. This experiment was terminated when the maximum tumor size reached ~1.5 cm in diameter. Euthanasia was performed after deep anesthesia to alleviate suffering and tumors were isolated from the animals and weighed.

### RNA-Seq

For each sample, a minimum of 3 μg of total RNA was oligo (dT) selected using the Dynabeads mRNA purification kit (Invitrogen, USA). Sequencing libraries were generated using NEBNext® UltraTM RNA Library Prep Kit for Illumina® (NEB, Ipswich, MA, USA) following manufacturer’s recommendations. Index codes were added to attribute sequences. Briefly, mRNA was purified from total RNA using poly-T oligo-attached magnetic beads. Double-stranded cDNA was synthesized, end-repaired, ligated to Illumina adapters, size selected on agarose gel (~250 bp) and PCR amplified. The cDNA library was sequenced on an Illumina HiSeq 6000 sequencing platform (BerryGenomics, Beijing, China). The gene expression levels for each transcript were estimated as the number of reads per kilobase of exon model per million mapped reads (RPKM) using only uniquely mapped reads in exonic regions. A gene is considered significantly differentially expressed if its expression differs between any two samples with the fold change > 2 and the *P*-value < 0.05 as calculated by Cufflinks. The RNA-Seq data are available at the Gene Expression Omnibus (GEO) (http://www.ncbi.nlm.nih.gov/geo/) under accession ID GSE173905, and GSE173906.

### Kyoto Encyclopedia of Genes and Genomes (KEGG) enrichment analysis

KEGG analysis was performed using KEGG database for pathways of differentially expressed genes to understand high-level functions and utilities of the biological system from molecular-level information (http://www.genome.jp/kegg/). Cluster Profiler R package was used to test the statistical enrichment of differential expression genes in KEGG pathways.

### Fatty acid analyses

Lipids were extracted by the Folch method and followed by alkaline hydrolysis to liberate fatty acids. For identification and relative quantification of fatty acids, chemical derivatization of samples spiked with internal standard was performed using *N*-(4-aminomethylphenyl) pyridinium (AMPP) (AMP + MaxSpec Kit, Cayman Chemical, MI, USA). AMPP-derivatized fatty acids were analyzed by reverse-phase (RP) liquid chromatography mass spectrometry (LC-MS). For determination of C = C locations in fatty acids, C = C-specific derivatization based on Paternò–Büchi reaction was conducted offline. The reaction solution was collected in vials for subsequent reversed-phase liquid chromatography (RPLC)-MS analysis. All LC-MS analyses were conducted on a LC-20AD system (Shimadzu, Kyoto, Japan) connected with an X500R QTOF mass spectrometer (Sciex, Toronto, CA).

### Immunohistochemistry (IHC)

Anti-Ki67 (1:500, 27309-1-AP, Proteintech) and anti-4-HNE (1:400, ab46545, Abcam) was used for immunohistochemistry. For immunohistochemistry, tumors isolated from animals were fixed in 4% v/v paraformaldehyde in PBS. Fixed tumors were equilibrated as follow: ethanol (70%) and ethanol (80%) for 90 min at 45 °C, respectively; 95% and 100 % v/v ethanol for 2 h each (three times) at 45 °C; xylene for 90 min at 45 °C (two times), and finally in paraffin for 2 h at 45 °C (three times). Samples were embedding in paraffin and the paraffin immersed in cold H_2_O and prepared 5 × 10^−6^ m sections by microtome. Then the sections were placed on charged glass slides distilled in H_2_O at 45 °C and dry overnight at room temperature (25-30°C). Slides were baked for 75 min at 60 °C, deparaffinized in xylene for 7 min (twice), rehydrated through 100% ethanol (twice, 5 min), 95% ethanol (5 min), 80% ethanol (5 min), 70% ethanol (5 min) and washed with PBS for three times, and then incubated for 30 min in methanol/H_2_O_2_ (0.9%) solution to quench endogenous peroxidases. Antigen retrieval was performed using citrate buffer at pH 6.0 for 10 min and then the slides were placed in a microwave for 30 s at 123 °C, and then were rinsed in distilled water for 2 min. Endogenous peroxidase activity was blocked in Peroxidazed (PX968M, BioCare Medical) for 8 min. The endogenous mouse IgG was blocked in Rodent Block (RBM961, BioCare Medical) for 20 min. The slides were then stained with the anti-Ki67 or without antibodies as negative controls for 1 h at room temperature (25–30°C), followed by rinsing in Tris-buffered saline containing Tween-20 (TBST). The slides were then treated with Rabbit-on-Rodent HRP Polymer (RMR622H, BioCare Medical) for 30 min and were incubated with the Betazoid DABKit (BioCare Medical) for 5 min in the dark, followed by rinsing with distilled water. Immunostained slides were counterstained with hematoxylin (Sigma-Aldrich, USA). Slides were scanned with ZEISS AxioScan Z1 and the number of positive cells per square millimeter was automatically calculated in QuPath software. Representative regions were selected to match the average density of positive cells in the sample.

### Statistical analysis

All in vitro experiments were performed in triplicate and repeated three times. Differences between variables were assessed by two-tailed Student’s *t*-test or one-way analysis of variance (ANOVA). Kaplan–Meier analysis was used to calculate the *P*-values of the survival curve. Log-rank analysis was used to calculate the median survival of mice in each group. The SPSS software 13.0 or GraphPad Prism version 8.0.1 statistical software package was used to perform all statistical analyses. The statistical data were represented as mean ± SD. In all assays, a *P-*value < 0.05 was considered statistically significant.

## Results

### Synergy of CB1 antagonists with ferroptosis inducers significantly inhibited TNBC cells growth

**Fig. 1:**
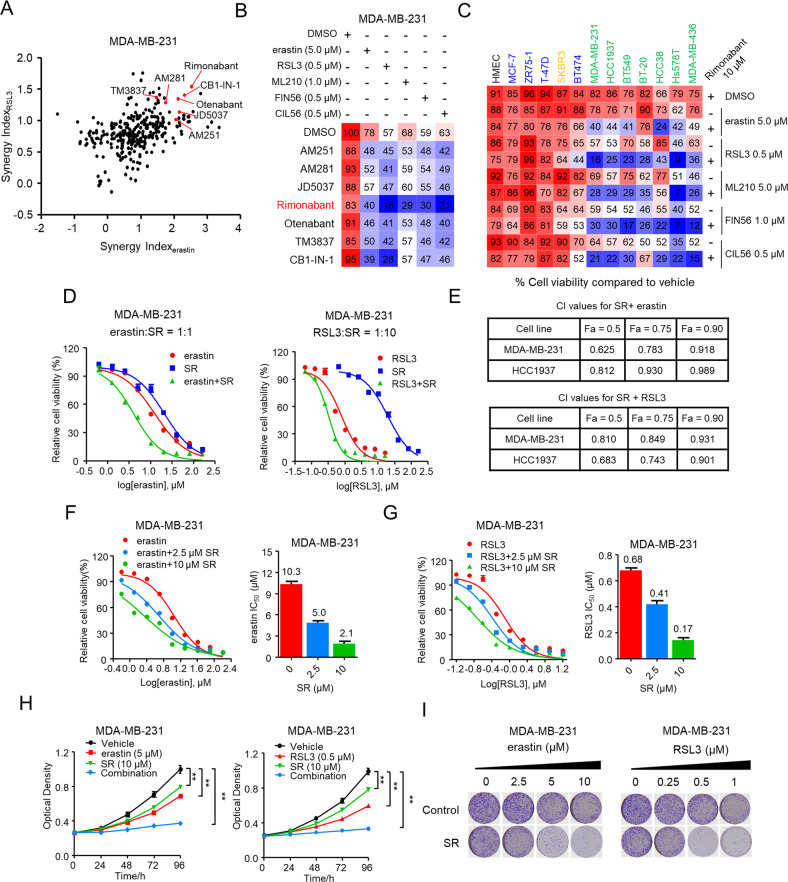
Synergy of CB1 antagonists with ferroptosis inducers significantly inhibited TNBC cells growth. **A** Volcano plot showing the results of drug screen in MDA-MB-231 cells. Synergy Index_(erastin)_ and Synergy Index_(RSL3)_ representing the combinational strength of 303 different lipid metabolism compounds with erastin and RSL3, respectively, for 72 h. Significant combinational treatment of CB1 antagonists with erastin and RSL3, respectively, are shown in red. **B** Combinational effects of CB1 antagonists (10 μM) with ferroptosis inducers (erastin, RSL3, ML210, FIN56, CIL56) at the indicated concentration treated with the inhibitors of single agent or the indicated target pairs in MDA-MB-231 cells. Viability was measured 72 h after treatment with the indicated concentrations of drugs. Effects on cell viability were calculated as percentage of vehicle-treated cells. **C** Combinational effects of rimonabant (SR) with ferroptosis inducers (erastin, RSL3, ML210, FIN56, CIL56) in the indicated breast cell lines, including normal-like cells (black), four luminal cell lines (blue), HER2-enriched cell line (orange) and 7 TNBC cell lines (green) were treated with the Inhibitors of single agent or the indicated target pairs. Viability was measured 72 h after treatment with the indicated concentrations of drugs. Effects on cell viability were calculated as percentage of vehicle-treated cells. **D**, **E** Effects of erastin or RSL3 with rimonabant (SR) as single agents or drug combinations in the indicated cell lines..... MDA-MB-231 cell viability was measured 72 h after treatment with the indicated doses of the drugs (**D**). Combinational Index (CI) was calculated by the Chou-Talalay equation using multiple doses and response points. CI values for three different indicated Fa are shown (**E**). **F**, **G** Dose-response curves erastin (**F**) or RSL3 (**G**) as single agents or drug combination with rimonabant (SR) at the indicated doses in the MDA-MB-231 cells for 72 h. The effects of SR on the IC_50_ of erastin or RSL3 are shown in the bar graphs (right). **H** The proliferation curve of single agents and drug combinations in the MDA-MB-231 cells treated with indicated concentrations of erastin and SR (left) or RSL3 and SR (right) for 96 h. The cell viability was assessed by CCK-8 assay treated with the indicated doses of the drugs. **p < 0.01 (one-way ANOVA). **I** In colony formation assays, MDA-MB-231 cells were treated with rimonabant (SR, 10 μM) or vehicle combined with the increasing concentrations of erastin (left) or RSL3 (right) for 14 days. Data shown are mean ± SD of triplicate measurements that were repeated three times with similar results.

As classical inducers of ferroptosis, erastin and RSL3, which inhibits system xc^-^ and glutathione peroxidase 4 (GPX4), respectively, are being widely explored as potential therapeutics various types of cancer cells [23, 24]. To explore the synergistic effect of co-targeting ferroptosis and lipid metabolism, we investigated the combinational effect erastin/RSL3 with 303 pharmacological compounds regulating lipid metabolism on growth of MDA-MB-231 and HCC1937 cells with unbiased screen. Out of the combination pairs we tested, we found that dual treatment of CB1 antagonists (AM251, AM281, JD5037, Rimonabant, Otenabant, TM3837, CB1-IN-1) with erastin/RSL3 showed higher combinational inhibition compared to single treatment as those compound Synergy Index_erastin_/Synergy Index_RSL3_ > 1, which reflected the potential synergistic effect (Fig. 1A and Fig. S1A). Next, we validated the screen results by combining CB1 antagonists with ferroptosis inducers including erastin, RSL3, ML210, FIN56, and CIL56. Importantly, among these combinational pairs, rimonabant exhibited the strongest effect in decreasing the viability of MDA-MB-231 and HCC1937 cells (Fig. 1B and Fig. S1B), which motivated further tests to investigate the synergic effect on TNBC inhibition.

We next assessed the combinational effects across multiple breast cell lines. As shown in Fig. 1C, rimonabant combined with ferroptosis inducers significantly triggered cell death in all TNBC cell lines we tested. In contrast, non-tumorigenic HMEC, luminal (MCF7, ZR75-1, T47-D, and BT474), and HER2-eriched (SKBR3) breast cancer cell lines were less sensitive to the combinations, indicating the unique vulnerability of TNBC cells to combinational therapies. The dose-response curve for rimonabant combined with erastin (rimonabant + erastin) or RSL3 (rimonabant + RSL3) revealed high potency and strong synergistic effect of drug synergy, as determined by the combination index using the Chou-Talalay method (combination index < 1; Fig. 1D, E and Fig. S1C–F). Conversely, no synergy was observed when rimonabant was combined with several first-line clinical TNBC drugs, including doxorubicin, taxol, carboplatin, and olaparib (combination index > 1; Fig. S2). In addition, compared with erastin or RSL3 single treatment, the rimonabant + erastin and rimonabant + RSL3 markedly reduced the IC_50_ values of TNBC cells (Fig. 1F, G and Fig. S3A–B) as well as markedly reduced cell proliferation and colony formation in TNBC cells (Fig. 1H, I and Fig. S3C, D). These above findings demonstrated the highly potent and specific synergy of rimonabant and ferroptosis inducers in inhibiting growth of TNBC cells.

### Rimonabant combined with erastin/RSL3 significantly inhibited TNBC cell growth by inducing ferroptosis and cell cycle arrest

**Fig. 2:**
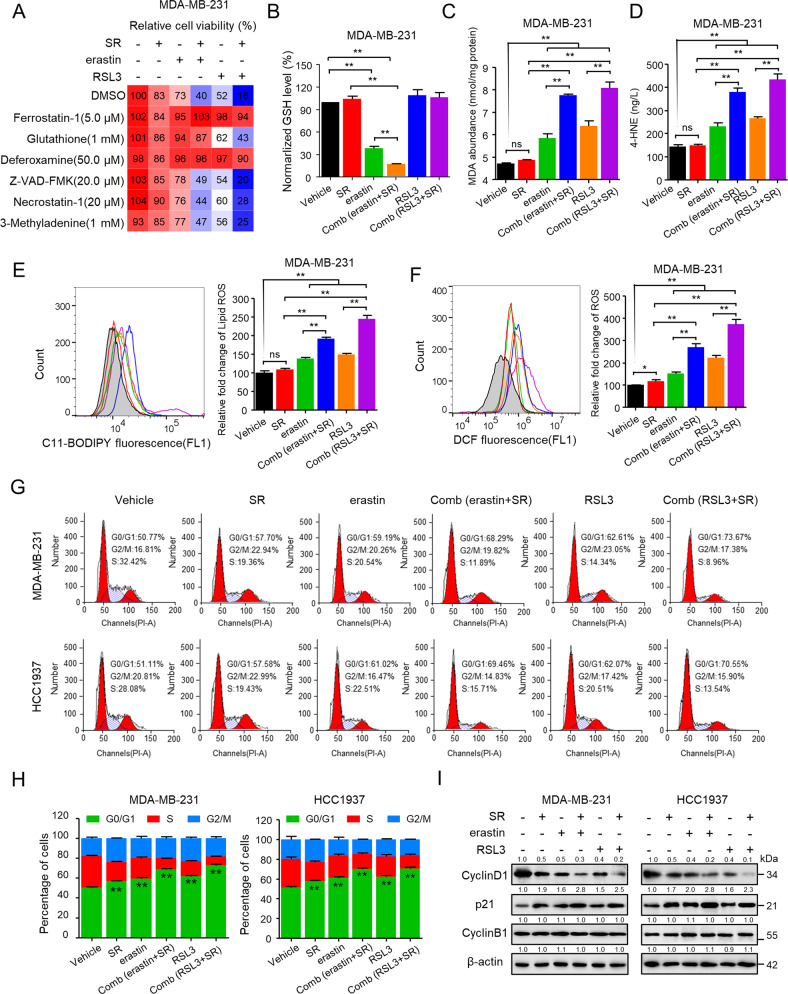
Rimonabant combined with erastin/RSL3 significantly inhibited TNBC cell growth by inducing ferroptosis and cell cycle arrest. **A** MDA-MB-231 cells were pretreated with the indicated cell death inhibitors for 1 h, and then cells were added with rimonabant (SR, 10 μM), erastin (5 μM) and RSL3 (0.5 μM) as single agents or drug combinations for an additional 72 h. Cell viability (CCK-8) is presented as percentage of untreated cells. **B** The level of GSH was determined after treated with rimonabant (SR, 10 μM), erastin (5 μM) and RSL3 (0.5 μM) as single agents or drug combinations for 48 h in MDA-MB-231 cells. **p < 0.01 (one-way ANOVA). **C**–**F** MDA (**C**), 4-HNE (**D**), lipid peroxidation (**E**) and cytosolic ROS (**F**) production of MDA-MB-231 cells treated as **B** Histograms show the production of MDA, 4-HEN, relative fold change of lipid ROS and cytosolic ROS (right panel). ns (no significance), *p < 0.05, **p < 0.01 (one-way ANOVA). **G**, **H** The cell cycle distributions of MDA-MB-231 and HCC1937 cells were treated as **B**. Cell cycle was evaluated by flow cytometry after staining with propidium iodide (PI). Histograms show the cell cycle distributions of treated MDA-MB-231 and HCC1937 cells **H**. **p < 0.01 versus the vehicle (*t*-test). **I** Representative western blotting assays of MDA-MB-231 and HCC1937 cells were treated as **B** with the indicated antibodies. Data shown are mean ± SD of triplicate measurements that were repeated three times with similar results.

We next explored underlying mechanisms alongside the reduction of cell viability by systematically examining the involvement of four major cell death pathways—apoptosis, necrosis, autophagy, and ferroptosis—using specific inhibitors. As seen in Fig. 2A and Fig. S4A, antioxidant Ferrostatin-1, GSH, and the iron chelator Deferoxamine, which are known to inhibit ferroptosis [25], could effectively reversed the effect of rimonabant + erastin and rimonabant + RSL3 on reducing cell viability. In contrast, the apoptosis inhibitor Z-VAD-FMK, necrosis inhibitor Necrostatin-1 as well as autophagic cell death inhibitor 3-methyladenine (3MA), respectively, only marginally rescued cell death in response to combinational treatment.

Then we investigated the combinational effect on ferroptosis characteristics including the level of lipid peroxidation, MDA, 4-HNE, cytosolic ROS level promotion and GSH depletion in TNBC cells. Intriguingly, though rimonabant itself did not exert GSH-depleting effect, whereas promoted the effect of erastin on GSH depletion (Fig. 2B and Fig. S4B). We also found that RSL3 as single agent or combined with rimonabant did not exert GSH-depleting effect, in consistent with study GSH remains unaffected with RSL3 treatment [26]. For the lipid peroxidation measurement, though rimonabant alone did not alter lipid peroxidation, MDA and 4-HNE level, it significantly promoted the effect of erastin/RSL3 on lipid peroxidation, MDA and 4-HNE induction compared with erastin/RSL3 single treatment (Fig. 2C–E and Fig. S4C–E). Moreover, combinational treatment markedly increased cytosolic ROS level compared with moderate increasing of single treatment (Fig. 2F and Fig. S4F).

We also evaluated those synergetic effect on the cell cycle in TNBC cells. Compared with the modest effect of rimonabant, erastin and RSL3 treated as single agent, combinational treatment exhibited remarkable effect on inducing G0/G1 cycle arrest (Fig. 2G, H). We also examined the expression of cell phase transition related proteins and genes expression. Rimonabant, erastin or RSL3 treated as single agent or their combination significantly reduced the expression of cyclin D1 and increased the level of p21, two major regulators of G1/S phase transition. However, neither the single agent nor the combinations altered the expression of cyclin B1, which is predominantly expressed during the G2/M phase (Fig. 2I). qPCR analysis revealed that mRNA expression of G0/G1 phase transition associated genes were significantly decreased such as CCND1, CCNE1, CDK4, CDK6 and etc, and CCKN1A, CCKN1B and GADD45A were significantly increased, whereas G2/M phase transition associated genes including CCNB1, CCNF, CCNA1 and CTSE1 did not exhibited dramatically changed (Fig. S4G). Taken together, these data verified that rimonabant combined with erastin/RSL3 significantly inhibited TNBC cell growth by inducing ferroptosis and cell cycle arrest.

### CB1 modulated the sensitivity of TNBC cells to ferroptosis

**Fig. 3:**
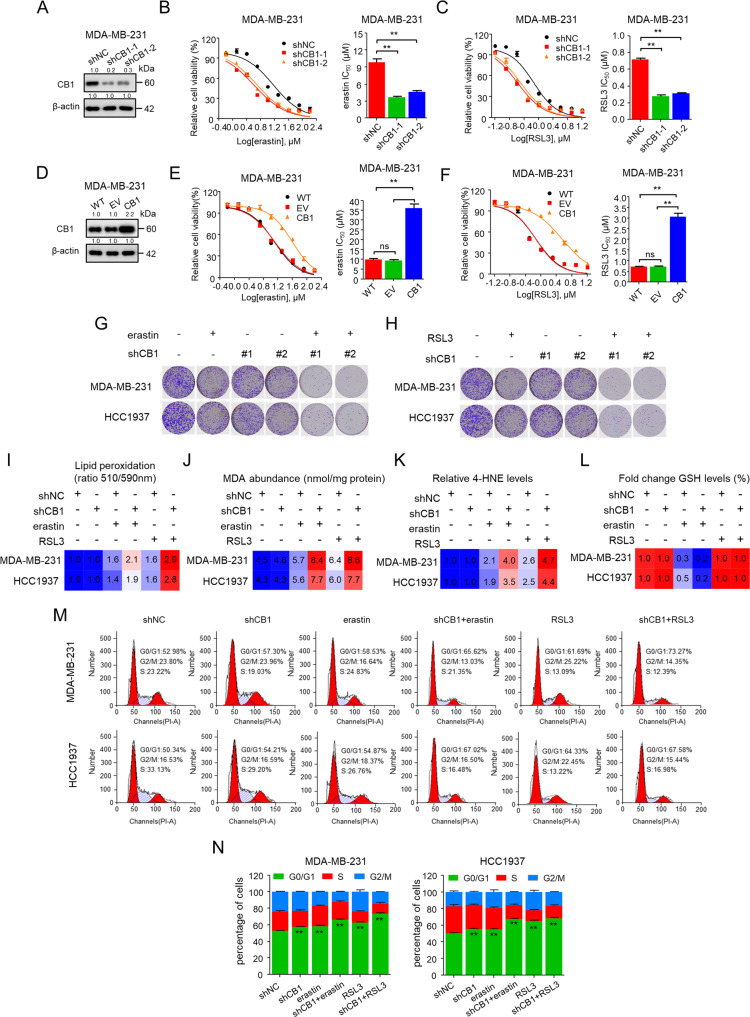
CB1 modulated the sensitivity of TNBC cells to ferroptosis. **A**–**C** Effect of CB1 knockdown on MDA-MB-231 cells in response to erastin/RSL3 treatment with two distinct CB1 shRNA expression vectors. WB analysis was conducted to detect CB1 expression in MDA-MB-231 cells that were stably transfected with negative control shRNA (shNC) or shCB1 **A**. Dose-response curves of MDA-MB-231 cell stably transfected with negative control vector or shCB1 were treated with erastin (**B**) or RSL3 (**C**) at the indicated doses. Cell viability was assessed by CCK-8 assay. The effects of negative control shRNA (shNC) or shCB1 on the IC_50_ of erastin or RSL3 are shown in the bar graphs (right). **p < 0.01 versus the corresponding control (*t*-test). **D**–**F** Effects of CB1 overexpression on MDA-MB-231 cells in response to erastin/RSL3 treatment. WB analysis was conducted to detect CB1 expression in parental MDA-MB-231 cells and cells that were stably transfected with empty vector (EV) or CB1 (**D**). Dose-response curves of MDA-MB-231 cell viability after stably transfected with empty vector or CB1 treated with erastin (**E**) or RSL3 (**F**) at the indicated doses. Cell viability was assessed by CCK-8 assay. The effects of empty vector, CB1 overexpression on the IC_50_ of erastin or RSL3 are shown in the bar graphs (right). ns (no significance), **p < 0.01 (one-way ANOVA). **G**, **H** In colony formation assays, MDA-MB-231 and HCC1937 cells stably transfected with negative control shRNA (shNC) or shCB1 treated with erastin (5 μM) (**G**) or RSL3 (0.5 μM) (**H**) for 14 days. **I**–**N** The lipid peroxidation (**I**), MDA (**J**), 4-HNE (**K**), GSH levels (**L**), and cell cycle distribution (**M**, **N**) of MDA-MB-231 and HCC1937 cells stably transfected with negative control shRNA (shNC) or shCB1 treated with erastin (5 μM) or RSL3 (0.5 μM). **p < 0.01 versus the vehicle (*t*-test) for cell cycle arrest (**M**, **N**). Data shown are mean ± SD of triplicate measurements that were repeated three times with similar results.

Next, we investigated whether CB1 modulates the sensitivity of TNBC cells to ferroptosis. Inhibition of CB1 using shRNAs strongly sensitized TNBC cells to erastin and RSL3, respectively (Fig. 3A–C and Fig. S5A–C). Conversely, when we overexpressed CB1, we found that it caused strong resistance reflected by markedly increased IC_50_ values of erastin- and RSL3-induced cell death (Fig. 3D–F and Fig. S5D–F). In addition, knockdown of CB1 significantly enhanced the effect of erastin and RSL3 on inhibiting cell colony formation (Fig. 3G, H). We next explored the effect of CB1 on regulating ferroptosis hallmarks and cell cycle phase transition. The data showed that CB1 knockdown enhanced the effect of erastin/RSL3 on increasing lipid peroxides, MDA and 4-HNE as well as the effect of erastin on GSH depleting (Fig. 3I–L). Moreover, CB1 knockdown combined with erastin/RSL3 strongly induced G1 cycle arrest (Fig. 3M, N). Taken together, these data proved that CB1 knockdown enhanced the sensitivity of TNBC cells to ferroptosis.

### CB1 regulated sensitivity of TNBC cells to ferroptosis by regulating SCD1- and FADS2-dependent fatty acid metabolism

**Fig. 4:**
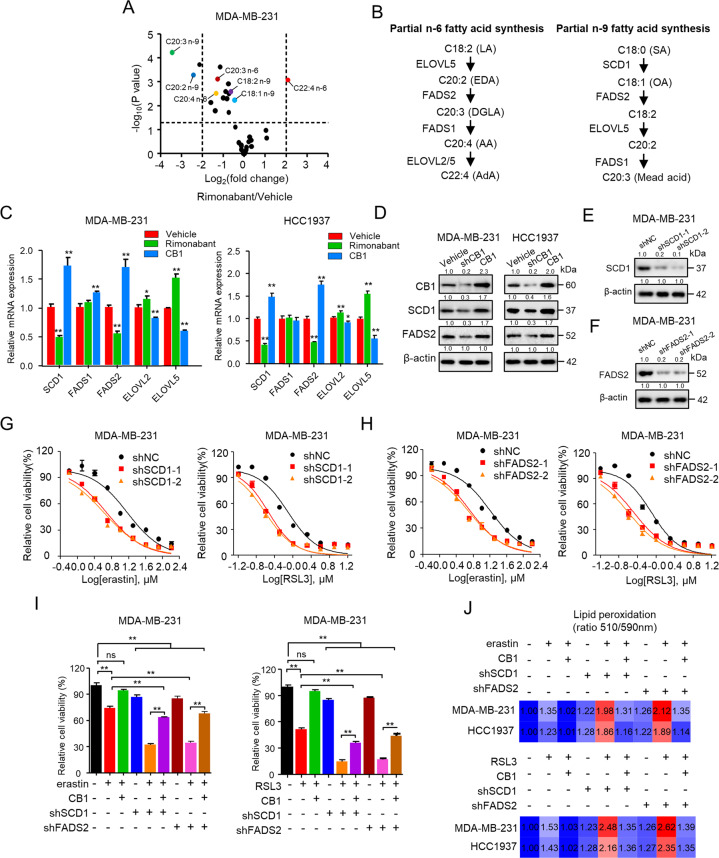
CB1 regulated sensitivity of TNBC cells to ferroptosis by regulating SCD1- and FADS2-dependent fatty acid metabolism. **A** Volcano plot showing fold changes and *P*-values for fatty acid species in rimonabant treated MDA-MB-231 cells compared with vehicle treatment. **B** Scheme showing the n-6 fatty acid and n-9 fatty acid partial synthesis pathway. n-6 fatty acid pathway: OA oleic acid, LA linoleic acid, EDA eicosadienoic acid, DGLA dihomo-γ-linolenic acid, AA arachidonic acid, AdA adrenic acid. n-9 FA pathway: SA stearic acid, OA, oleic acid; MA, mead acid. **C** qPCR analysis of the indicated gene expression associated with fatty acid metabolism in MDA-MB-231 and HCC1937 cells treated with rimonabant or CB1 overexpression. *p < 0.05, **p < 0.01 (*t*-test). **D** WB analysis was conducted to detect CB1, SCD1 and FADS2 in MDA-MB-231 and HCC1937 cells that were stably transfected with control vector, shCB1 or CB1. **E**–**H** Effect of SCD1 and FADS2 knockdown on MDA-MB-231 cells in response to erastin and RSL3 treatment, respectively, using two distinct target gene shRNA expression vectors. WB analysis was conducted to detect SCD1 (**E**) and FADS2 (**F**) in MDA-MB-231 cells that were stably transfected with negative control shRNA (shNC), shSCD1 or shFADS2. Dose-response curves of MDA-MB-231 cell stably transfected with negative control shRNA (shNC), shSCD1 (**G**) or shFADS2 (**H**) treated with erastin (left) or RSL3 (right) at the indicated doses. The cell viability was assessed by CCK-8 assay after treatment for 72 h with the indicated doses of the drugs. **I** The cell viability of MDA-MB-231 cells stably transfected with empty vector, CB1, shFADS2 was treated with erastin (5 μM) or RSL3 (0.5 μM). Cell viability was assessed by CCK-8 assay after treatment for 72 h with the indicated doses of the drugs. ns (no significance), **p < 0.01 (one-way ANOVA). **J** Effect of CB1 overexpression, SCD1 and FADS2 knockdown on lipid peroxidation response to erastin (up) and RSL3(down) treatment, respectively, in MDA-MB-231 and HCC1937 cells. Data shown are mean ± SD of triplicate measurements that were repeated three times with similar results.

PUFA biosynthesis plays an essential role in determining ferroptosis sensitivity [15, 27], which prompted us to hypothesize that fatty acid metabolism may participated in CB1 regulated ferroptosis. We first characterized alterations in fatty acid profiles caused by rimonabant treatment (Fig. S6). Previous studies have demonstrated that PUFA biosynthesis catalyzed by different desaturase enzymes can discriminate the cellular susceptibility to ferroptosis [12, 28], we thus analyzed fatty acid species level in rimonabant treated MDA-MB-231 cells compared with vehicle treatment to explore the enzymes that might associated with CB1 modulated ferroptosis sensitivity (Fig. 4A and Supplementary Table 4). We found that rimonabant treatment significantly increased the abundance of FA 22:4 n-6 (adrenic acid, AdA) but decreased the abundance of other n-6 fatty acids including FA 20:2 n-6 (eicosadienoic acid, EDA), FA 20:3 n-6 (dihomo-γ-linolenic acid, DGLA) and FA 20:4 n-6 (arachidonic acid, AA) as well as the levels of FA 20:2 n-9 and FA 20:3 n-9 (mead acid, MA). Previous studies have already reported the n-6 biosynthesis pathway of AdA regulated by desaturates such as FADS1, FADS2, and elongases like elongation of very long fatty acids protein 2/5 (ELOVL2/5); FA 20:2 and FA 20:3 n-9 biosynthesis regulated by desaturates SCD1, FADS1, FADS2 and elongase ELOVL5 [27, 29] (Fig. 4B). We then performed qPCR to estimate mRNA expression of the genes involved in these pathways. Rimonabant treatment resulted in upregulated mRNA of ELOVL5 which accounted for elevated FA 22:4 (AdA) and decreased mRNA of SCD1/FADS2 which accounted for downregulation of FA 20:2 and FA 20:3 n-9 (Fig. 4C), and there was no obvious change in FADS1 mRNA. Moreover, knockdown of CB1 decreased the protein expression of SCD1 and FADS2 in MDA-MB-231 and HCC1937 cells, in contrast to the upregulated effect of CB1 overexpression on SCD1 and FADS2 (Fig. 4D).

SCD1 and FADS2 are responsible for biosynthesis of monounsaturated fatty acids to inhibit ferroptosis in ovarian and breast cancer cells [28, 30, 31]. We next characterized the function of SCD1 and FASD2 in modulating the sensitivity of TNBC cells to ferroptosis and whose role in CB1 regulated ferroptosis sensitivity. Knockdown of SCD1 and FASD2, respectively, using shRNAs enhanced the sensitivity of TNBC cells to erastin and RSL3 (Fig. 4E–H and Fig. S7A–D). Intriguingly, inhibition of SCD1 and FASD2 by shRNAs abolished the ability of CB1 to activate SCD1 and FASD2 (Fig. S7F) and partially abolished the ability of CB1 in repressing the inhibitory effect of erastin/RSL3 in MDA-MB-231 and HCC1937 cells (Fig. 4I and Fig. S7G). We also measured the effect of CB1 overexpression, SCD1 and FADS2 knockdown on lipid peroxidation. As shown in Fig. 4J, SCD1 and FADS2 knockdown significantly increased the levels of lipid peroxidation in response to erastin/RSL3 treatment, and inhibition of SCD1 and FASD2 attenuated the effect of CB1 on repressing erastin/RSL3 induced lipid peroxidation accumulation in MDA-MB-231 and HCC1937 cells. These data suggested that SCD1 and FADS2 were involved in CB1 regulated ferroptosis sensitivity in TNBC cells.

### PI3K-AKT and MAPK pathways were involved in CB1 repressed the ferroptosis sensitivity

**Fig. 5:**
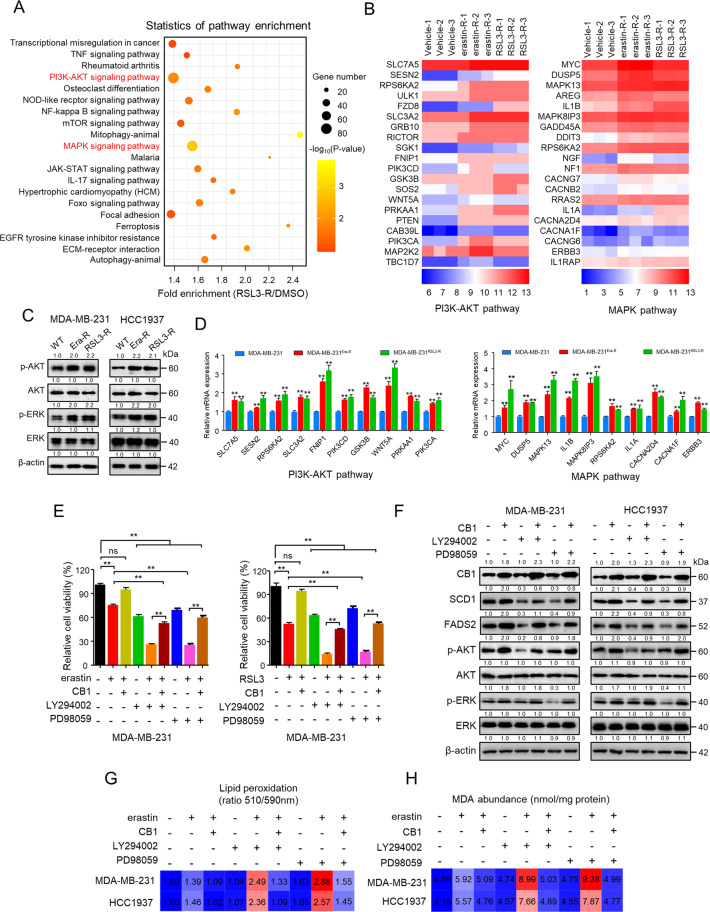
PI3K-AKT and MAPK pathways were involved in CB1 repressed the ferroptosis sensitivity. **A** KEGG pathway analysis of differentially expressed genes in MDA-MB-231 cells transcriptome surviving from increased RSL3 treatment (RSL3-R) (The top 20 most significantly activated pathway are shown). **B** Heatmap of significantly regulated genes of MDA-MB-231 cells transcriptome surviving from increased erastin treatment (erastin-R) and RSL3 treatment (RSL3-R), respectively, correlated with PI3K-AKT pathway (left) and MAPK pathway (right) (*n* = 3). **C** WB analysis of MDA-MB-231 and HCC1937 cells that were resistant to erastin (Era-R) and RSL3(RSL3-R), respectively. **D** qPCR analysis of the indicated gene expression associated with PI3K-AKT pathway (left) and MAPK pathway (right) in MDA-MB-231 cells that were resistant to erastin (MDA-MB-231^Era-R^) and RSL3 (MDA-MB-231^RSL3-R^), respectively, compared with parental MDA-MB-231 cells. **p < 0.01 (*t*-test). **E** The cell viability of MDA-MB-231 cells stably transfected with empty vector or CB1 treated with LY294002 (5 μM) or PD98059 (10 μM). The cell viability was assessed by CCK-8 assay after treatment for 72 h with the indicated doses of the drugs. ns (no significance), **p < 0.01 (one-way ANOVA). **F** WB analysis of MDA-MB-231 and HCC1937 cells transfected with empty vector or CB1. Cells were treated with LY294002 (5 μM) or PD98059 (10 μM). **G**, **H** Effect of CB1 overexpression, LY294002 (5 μM) and PD98059 (10 μM) on lipid peroxidation (**G**) and MDA (**H**) response to erastin treatment, respectively, in MDA-MB-231 and HCC1937 cells. Data shown are mean ± SD of triplicate measurements that were repeated three times with similar results.

To systematically investigate potential mechanisms involving in modulating ferroptosis sensitivity and the pathway underlying CB1 regulated ferroptosis, genome-wide RNA sequencing was performed to identify pro-ferroptosis resistance genes in MDA-MB-231 cells surviving from increased concentration treatment of erastin and RSL3, respectively (Fig. S8A–D). KEGG pathway analysis of the overlapped 226 genes revealed that the PI3K-AKT and MAPK pathways were significantly hyperactive (Fig. 5A, B and Fig. S8E, F), indicating that hyperactive PI3K-AKT and MAPK pathways are involved in contributing to ferroptosis-resistant state of TNBC cells and regulating ferroptosis sensitivity. To test this simple hypothesis, we generated MDA-MB-231 and HCC1937 cell lines intrinsically resistant to erastin/RSL3, in which the IC_50_ values for erastin or RSL3 were increased by >10-fold (Fig. S9A, B). Notably, we found that the expression of phosphorylated AKT and ERK was increased in erastin/RSL3-resistant TNBC cells (Fig. 5C) as well as mRNA level of genes related to the PI3K-AKT and MAPK pathways (Fig. 5D and Fig. S9C), suggesting their role in regulating ferroptosis sensitivity in TNBC cells. We also identified that the expression of CB1 was increased in erastin-/RSL3-resistant MDA-MB-231 and HCC1937 cells (Fig. S10A). Moreover, we explored the combination therapy in erastin-/RSL3-resistant TNBC cells. Intriguingly, erastin (50 μM) and RSL3 (5 μM) showed no inhibitory effect in erastin-/RSL3-resistant MDA-MB-231 and HCC1937 cells, however, synergy of rimonabant with erastin/RSL3 significantly reduced erastin-/RSL3-resistant MDA-MB-231 and HCC1937 cell viability (Fig. S10B, C). We also evaluated the level of lipid peroxidation upon combination treatment in erastin-/RSL3-resistant cells. As shown in Fig. S10D, E, rimonabant, erastin or RSL3 treated as single agent had no effect on inducing lipid peroxidation, however, rimonabant synergized with erastin/RSL3 increased lipid peroxidation production in erastin-/RSL3-resistant TNBC cells compared with monotherapy, indicating that rimonabant can reverse the resistant state of TNBC cells to ferroptosis. We next explored whether CB1 represses ferroptosis sensitivity through activation of AKT and ERK. Firstly, inhibition of ERK1/2 and AKT by PD98059 and LY294002, respectively, partially abolished the ability of CB1 to repress the inhibitory effect of erastin/RSL3 on decreasing MDA-MB-231 and HCC1937 cells viability (Fig. 5E and Fig. S10F). In addition, inhibition of ERK1/2 and AKT by PD98059 and LY294002, respectively, abolished the ability of CB1 to activate ERK and AKT, and the ability of CB1 to enhance the expression of SCD1 and FADS2 can be abrogated by inhibition of AKT/ERK (Fig. 5F). Moreover, PD98059 and LY294002 enhanced the effect of erastin/RSL3 on lipid peroxidation and MDA production, which were attenuated by CB1 overexpression (Fig. 5G, H and Fig. S10G, H). Taken together, our data suggested that CB1 regulated SCD1 and FADS2-associated fatty acid metabolism to modulate the sensitivity of TNBC cells to ferroptosis via the AKT and ERK pathway.

### Rimonabant combined with erastin/RSL3 significantly repressed TNBC tumor growth in vivo

**Fig. 6:**
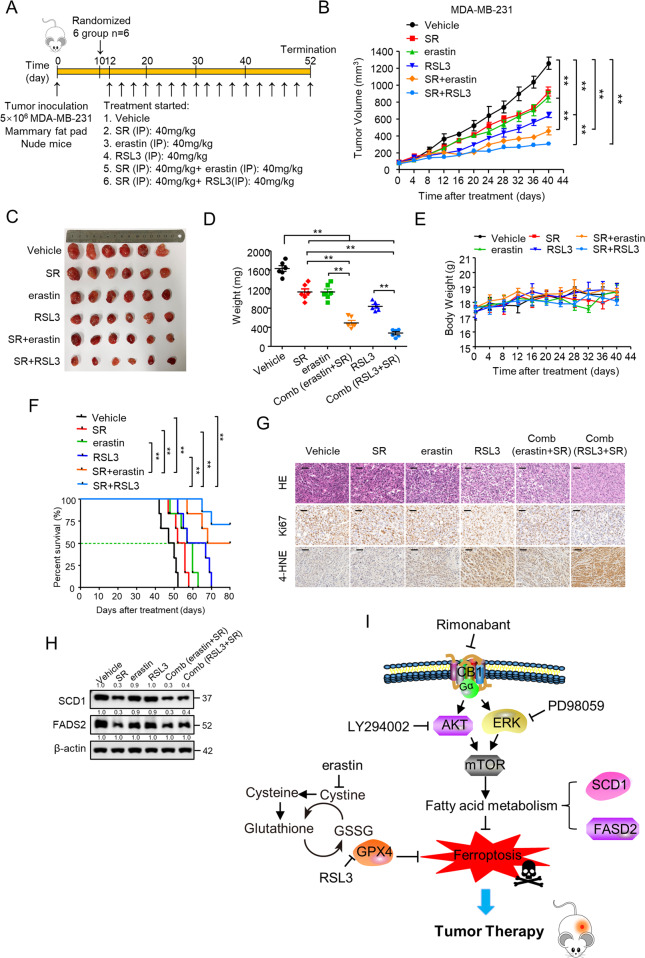
Rimonabant combined with erastin/RSL3 significantly repressed TNBC tumor growth in vivo. **A** Treatment schedule for the MDA-MB-231 cells orthotopic implantation model was treated with vehicle, rimonabant (SR), erastin and RSL3 as single agents or drug combinations. **B**–**D** Mice were treated as (**A**) 12 days after xenograft, and tumor size was monitored every other day **B**. Photograph of tumors (**C**) and tumor weight (**D**) are shown (*n* = 6 per group). **p < 0.01 (one-way ANOVA). **E** Body weight of BALB/c nude mice treated as (**A**) were shown (*n* = 6 per group). **F** Survival rates for nude mice treated as in (**A**) were shown (log-rank test; *n* = 6 per group). **p < 0.01 (Kaplan–Meier). **G** Representative images of H&E, Ki-67 and 4-HNE IHC staining in harvested tumors from each group were shown. Scale bars represent 50 mm. **H** WB analysis of SCD1 and FADS2 in harvested tumors from each group were shown. **I** Proposed model for CB1 regulated SCD1- and FADS2-dependent fatty acid metabolism via PI3K-AKT and MAPK signaling pathways to inhibit TNBC cell growth. Data shown are the means ± SD from six tumors at each time point.

We further examined the combinational effect on MDA-MB-231 and HCC1937 tumor growth in nude mice by assessing the effects of rimonabant, erastin, and RSL3 as single agents or their combinations (Fig. 6A and Fig. S11A). As shown, rimonabant (40 mg/kg) and erastin (40 mg/kg) applied as a single agent led to a modest tumor growth inhibition (TGI)% values of 27.6% and 32.3%, respectively, in MDA-MB-231 xenograft tumor, whereas rimonabant + erastin combination was very effective and drastically reduced tumor size, with a TGI% values of 76.5%. In addition, rimonabant + RSL3 led to a TGI% values of 84% compared with 49.1% of RSL3 (40 mg/kg) treated as monotherapy (Fig. 6B–D). Similarly, rimonabant (40 mg/kg), erastin (40 mg/kg) and RSL3 (40 mg/kg) monotherapy led to a TGI% values of 31.2%, 39.9% and 50.2% in HCC1937 xenograft tumor, respectively, whereas rimonabant + erastin and rimonabant + RSL3 led to a TGI% values of 78.8% and 88.5% (Fig. S11B–D), indicating the significant inhibitory effect on tumor growth of the combination treatment. These drug combinations had no obvious toxicity at the applied doses, as indicated by the slight floating body weight during the experiment (Fig. 6E and Fig. S11E). Kaplan–Meier survival curves of nude mice with MDA-MB-231 xenograft tumor showed that vehicle-treated, rimonabant, erastin and RSL3 monotherapy showed no difference in median or durable survival. In contrast, combinational treatment resulted in a significant durable survival advantage over vehicle and single agent treatment (Fig. 6F). Additionally, median survival of tumor-bearing animals was increased with rimonabant + erastin (74 vs. 48 days) and rimonabant + RSL3 ( > 80 vs. 48 days) treatment, indicating that rimonabant enhances the effect of erastin/RSL3 on improving survival. Furthermore, the images of hematoxylin and eosin (H&E)-stained tumor tissues, Ki67-stained and lipid peroxide 4-HNE-stained immunohistochemical sections of the tumors showed that the number of tumor foci in the combinational treatment group was much lower than that in the empty vector or single agent group, and combination groups showed a more significant inhibitory effect on the amount of Ki67 positive cells and significantly increased expression of 4-HNE. (Fig. 6G and Fig. S11F), confirming their profound inhibitory effect on TNBC tumor growth via ferroptosis induction. As expected, the tumors in mice with rimonabant and combinational treatment had reduced levels of SCD1 and FADS2 (Fig. 6H and Fig. S11G). Finally, the effect of CB1 knockdown on the sensitivity of TNBC cell growth to ferroptosis was investigated. MDA-MB-231 cells stably infected with CB1 shRNA lentivirus or empty vector, or parental MDA-MB-231 cells were injected subcutaneously in nude mouse which were then treated with erastin or RSL3 (Fig. S12A). Compared with erastin and RSL3 monotherapy, knockdown of CB1 significantly enhanced the effect of erastin/RSL3 on suppressing TNBC cells growth (Fig. S12B–F). Taken together, these results suggest that co-targeting of CB1 and ferroptosis could be a promising therapeutic approach for TNBC.

## Discussion

In this study, drug combination screen was utilized to explore synergistic therapies of dual targeting lipid metabolism and ferroptosis in TNBC cells. We found that CB1 antagonist rimonabant combined with ferroptosis inducers showed potent inhibitory efficiency and high specificity in decreasing TNBC cell viability via inducing ferroptotic cell death and G1 cell cycle arrest. Mechanically, through RNA-Seq, fatty acid analyses and functional studies, we discovered that CB1 repressed the sensitivity of TNBC cells to ferroptosis by regulating SCD1-, FADS2-regulated fatty acid metabolism via PI3K-AKT and MAPK pathways. In vivo evaluation by using orthotopic tumor models manifested strong synergy of rimonabant with erastin/RSL3 in decreased TNBC tumor growth. Moreover, rimonabant can reverse the resistant state of TNBC cells to erastin/RSL3. (Fig. 6I). Our results demonstrated that synergy of CB1 antagonists with ferroptosis inducers could be a promising therapeutic strategy for TNBC.

As one of the most important metabolic pathways, lipid metabolism has been shown to play an essential role in regulating ferroptosis sensitivity [15, 16]. As reflected by the systematic analysis of the drug combination screen of lipid metabolism with ferroptosis inducers, a variety of pharmacological compounds including HMG-CoA reductase inhibitors, AMPK inhibitors, ATP-citrate lyase (ACLY) inhibitors, cytochrome P450 (CYP450) inhibitors and SREBP inhibitors were identified to exhibit promising synergistic effects with erastin or RSL3 (Supplementary table 2), whose roles in directly or indirectly regulating ferroptosis sensitivity have already been elucidated [12, 14, 32]. Intriguingly, other pharmacological compounds such as CB1 antagonists, PPAR antagonists, FAAH inhibitors, CEPT inhibitors have not been elucidated modulate ferroptosis. Rimonabant, a CB1 antagonist, has been developed for the clinical treatment of obesity and its metabolic complications including regulating lipid metabolism. Increasing evidence have demonstrated the potential of targeting CB1 as an anticancer strategy because of its pivotal role in cancer development and progression [33, 34] and accumulating studies have also verified the inhibitory effect of rimonabant in various malignancies, including breast cancer [35], colon cancer [36, 37], and fibrosarcoma [38]. In this study, though rimonabant treated as single agent showed no effect on ferroptosis, it synergized with erastin/RSL3 significantly reduced cell viability and increased lipid peroxidation production compared with monotherapy. What is more, rimonabant can reverse the resistant state of TNBC cells to ferroptosis in erastin-/RSL3-resistant TNBC cells, highlighting the role of lipid metabolism reprogramming in regulating ferroptosis sensitivity. Though rimonabant was withdrawn from the market because of its risk of neuropsychiatric adverse events, other CB1 antagonists such as Drinabant, Rosonabant, AVE-1625 and TM38837, are being developed in clinical trials that specifically act on CB1 in peripheral tissues, which are unlikely to penetrate the blood-brain barrier, thereby minimizing central side effects [39]. Thus, further effort should be made for systematic synergy test of rimonabant combined with ferroptosis inducers to identify potent inhibitory effect without central side effect for TNBC therapy and the combinational effect of other CB1 antagonists with ferroptosis induces.

Previous studies have demonstrated monounsaturated fatty acids (MUFA) and PUFA biosynthesis catalyzed by different desaturase enzymes resulting in discriminating the cellular susceptibility to ferroptosis [12, 28]. Studies have shown that genetic or pharmacologic blockade SCD1 enhances sensitivity to ferroptosis in ovarian cancer and SREBP1 protects cells from ferroptosis through SCD1 activity [40, 41]. FADS2 knockdown significantly increases the levels of iron and lipid ROS and decreases the levels of ferroptosis-related genes, which ultimately induces ferroptosis to suppress growth of lung tumor [42]. As shown in Fig. S7E, compared with normal cells, luminal or HER2-enriched breast cancer cells, TNBC cells exhibited higher SCD1 and FADS2 expression compared with, which may explain their high sensitivity to combinational treatment of CB1 and ferroptosis inducers. Here, we also found that knockdown of SCD1 or FASD2 increased the sensitivity of TNBC cells to erastin or RSL3. Therefore, the inhibition of these enzymes combined with ferroptosis inducers will also be potentially useful for cancer treatment in the future.

In the last decade, numerous lipid metabolic pathways have been studied to regulating ferroptosis sensitivity, unfortunately, carcinoma cells that were initially sensitive to ferroptosis can also switched to a ferroptosis-resistant state which is associated with extensive downregulation of PUFA-ePLs [43]. Thus, the following question arises: which lipid metabolic pathways are involved in regulating ferroptosis sensitivity as well as evasion? Herein, RNA-Seq of TNBC cells surviving from increased treatment with erastin or RSL3 revealed diverse signaling pathways that may regulate ferroptosis sensitivity and facilitate ferroptosis evasion, including activation of PI3K-AKT and MAPK signaling pathways. Since CB1 has been shown to modulate Akt-mTORC1 (mechanistic target of rapamycin complex 1) and MAPK pathways in various types of cancer cells, resulting in affecting hallmarks of cancer [21]. Herein, we identified that PI3K-AKT and MAPK pathways were involved in CB1 regulated SCD1, FADS2 expression to repress the ferroptosis sensitivity. Previous studies have shown that activation of the PI3K-AKT and MAPK pathways transcription, translation, proliferation, growth and survival [44, 45], and PI3K-AKT and MAPK pathways act as most frequently activated drivers of human cancers by reprograming lipid metabolism [32, 46]. Recent study revealed the oncogenic activation of PI3K-AKT-mTOR signaling suppresses ferroptosis via SREBP-mediated lipogenesis [41]. In addition, multi-stage differentiation defines melanoma subtypes with differential vulnerability to ferroptosis distinguish dedifferentiation-associated resistance involving MAPK re-activation [47]. Thus, further effort should be conducted to identify the effect of combination of LY294002 and PD98059 with ferroptosis inducers on abrogating ferroptosis resistance. Apart from this, other signaling pathways such as TNF, NF-kappa B, JAK-STAT, Foxo, TGF-beta signaling pathways were also shown to be hyperactivated as reflected by KEGG analysis. TNF signaling has been shown to be relevant for breast cancer tumor progression and metastasis as well as acquired drug resistance [48]. It has recently been reported that TNF antagonist sensitizes synovial fibroblasts to ferroptotic cell death in collagen-induced arthritis mouse models [49]. Though the KEGG analysis also showed that the TNF signaling pathway was hyperactive in MDA-MB-231 cells surviving from increased concentration treatment RSL3, mRNA level of genes related to the TNF signaling pathway were partly increased RSL3-resistant TNBC cells but not increased in erastin-resistant TNBC cells (Fig. S9D), indicating that TNF signaling pathway might not be involved in regulating TNBC ferroptosis resistance. In spite of this, further investigation will be needed to explore the role of TNF signaling in regulating ferroptosis as well as other signaling pathways in more details.

## Supplementary information

Supplementary Figures

Supplementary Figure legends

Supplementary table 1

Supplementary table 2

Supplementary table 3

Supplementary table 4

Original full length WB

reproducibility checklist

## Data Availability

The rest datasets used or analyzed during the current study are available from the corresponding author on reasonable request.
