# Supplementary material for: Inhibition of cannabinoid receptor type 1 sensitizes triple-negative breast cancer cells to ferroptosis via regulating fatty acid metabolism
Source: Cell Death Dis. 2022 Sep 21;13(9):808. doi: 10.1038/s41419-022-05242-5 (PMC9492666; doi:10.1038/s41419-022-05242-5)

# Supplementary Figure 1

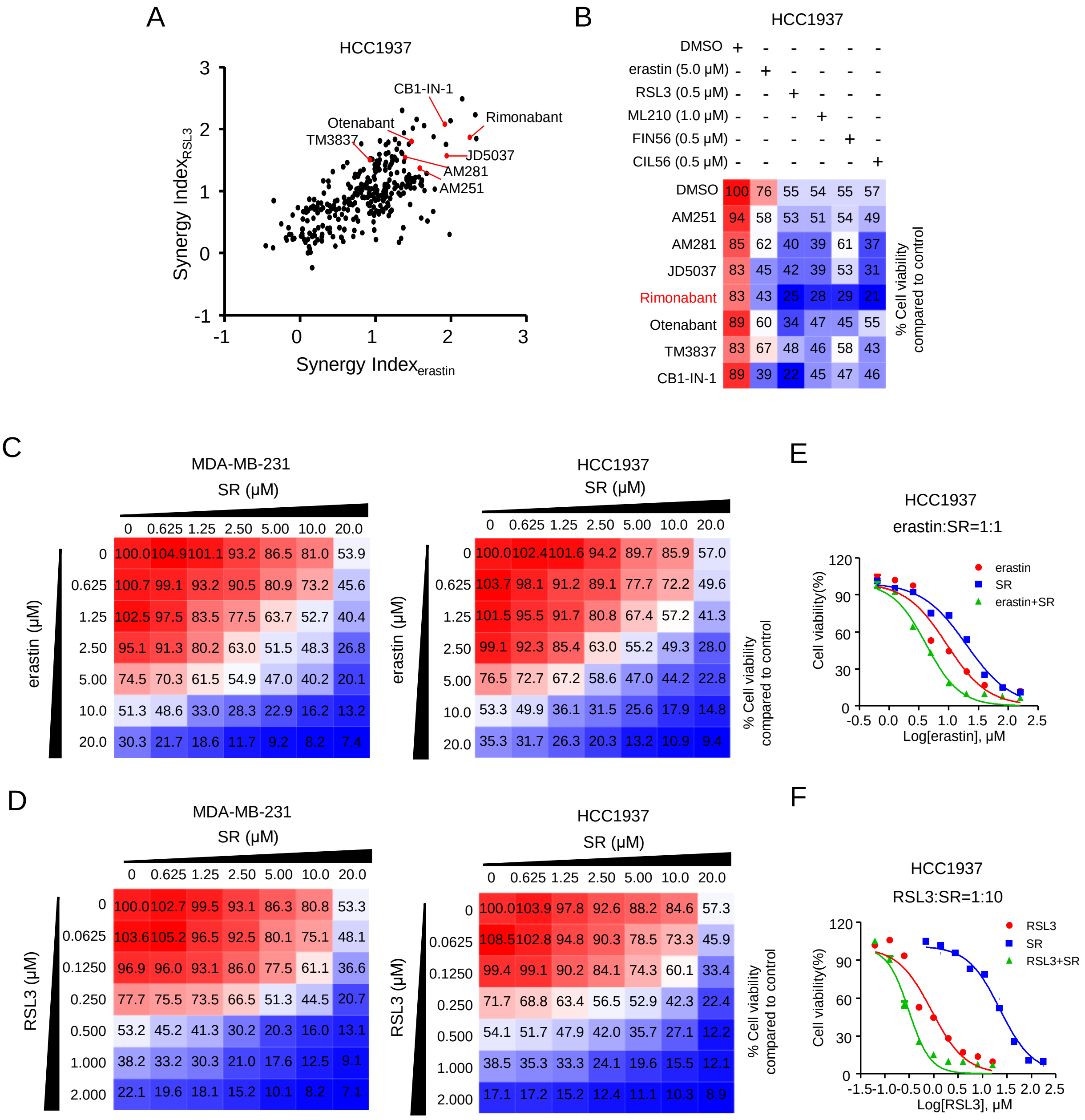

# Supplementary Figure 2

A

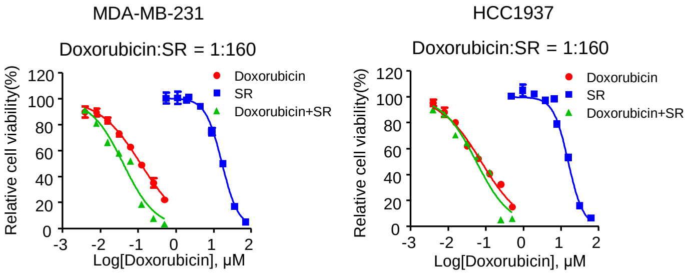


CI values for SR + Doxorubicin

| Cell line | Fa = 0.5 | Fa = 0.75 | Fa = 0.90 |
|---|---|---|---|
| MDA-MB-231 | 5.51 | 4.45 | 3.62 |
| HCC1937 | 6.18 | 8.61 | 12.98 |

B

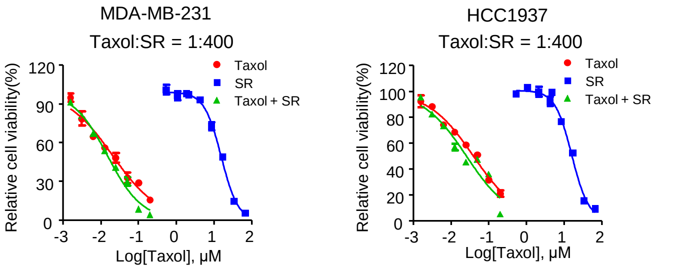


CI values for SR + Taxol

| Cell line | Fa = 0.5 | Fa = 0.75 | Fa = 0.90 |
|---|---|---|---|
| MDA-MB-231 | 8.25 | 6.81 | 5.81 |
| HCC1937 | 3.47 | 3.65 | 4.08 |

C

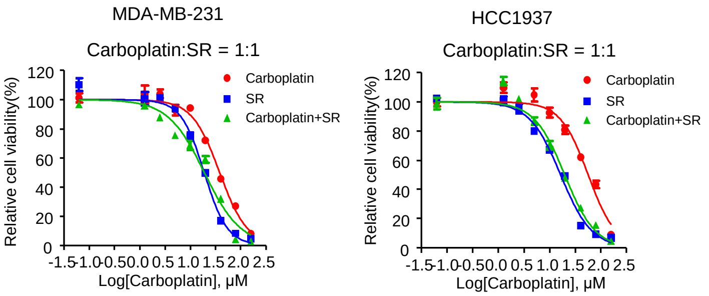


CI values for SR + Carboplatin

| Cell line | Fa = 0.5 | Fa = 0.75 | Fa = 0.90 |
|---|---|---|---|
| MDA-MB-231 | 3.39 | 2.84 | 2.38 |
| HCC1937 | 7.67 | 7.75 | 7.90 |

D

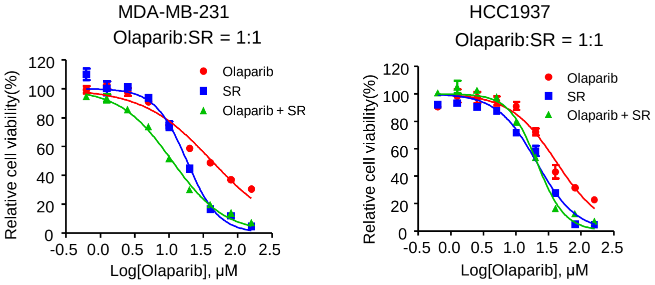


CI values for SR + Olaparib

| Cell line | Fa = 0.5 | Fa = 0.75 | Fa = 0.90 |
|---|---|---|---|
| MDA-MB-231 | 4.03 | 3.25 | 2.63 |
| HCC1937 | 7.36 | 5.21 | 3.69 |

# Supplementary Figure 3

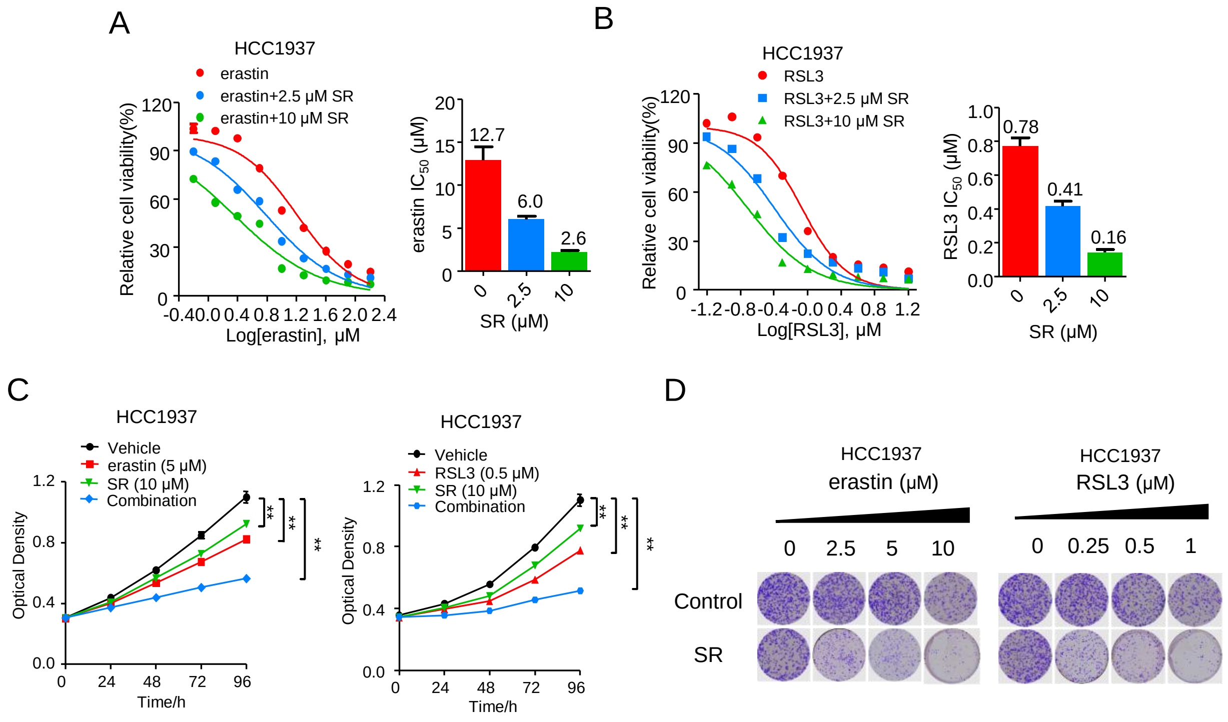

# Supplementary Figure 4

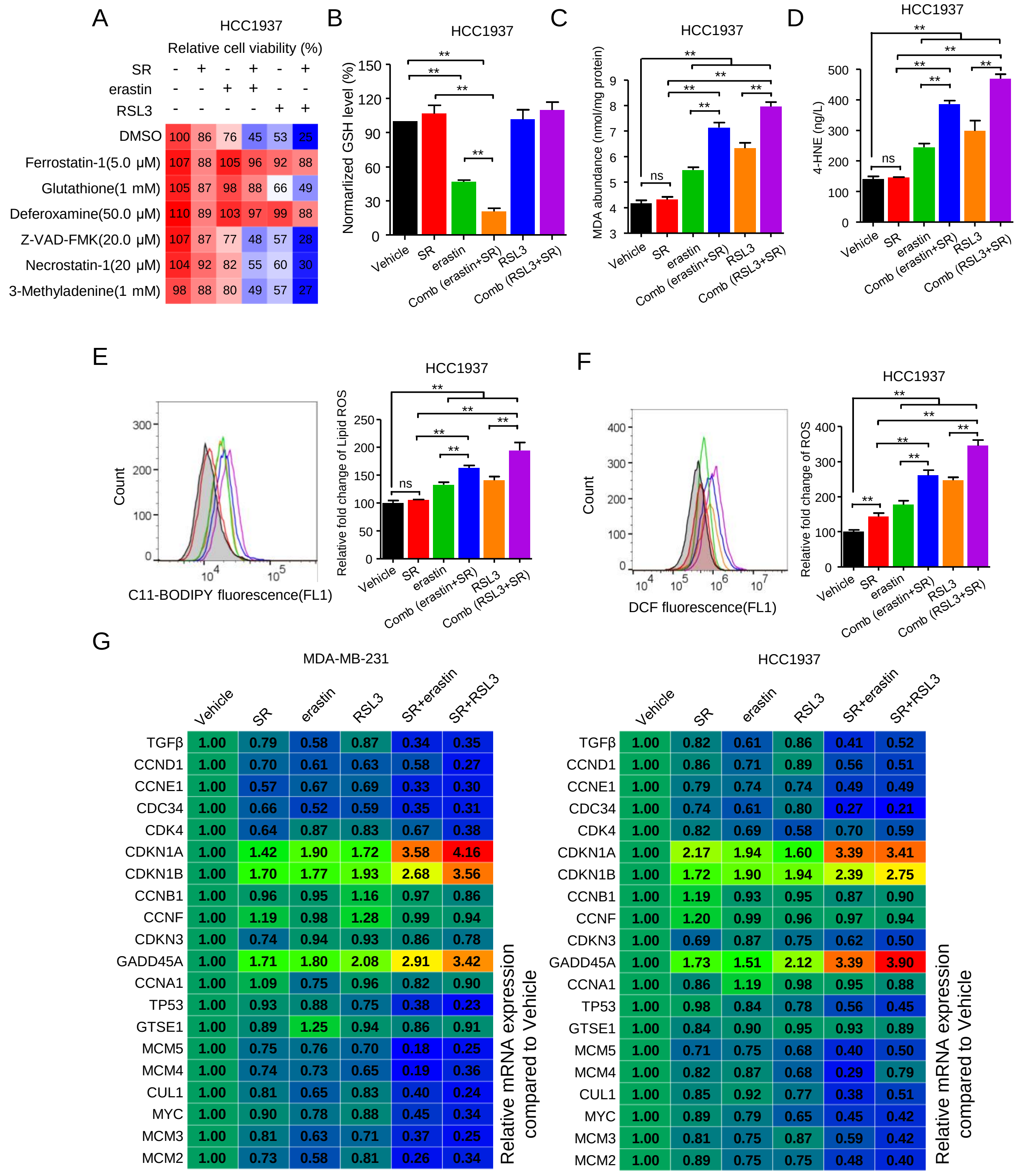


**A** HCC1937 — Relative cell viability (%)

| | SR - / erastin - / RSL3 - | SR + / erastin - / RSL3 - | SR - / erastin + / RSL3 - | SR + / erastin + / RSL3 - | SR - / erastin - / RSL3 + | SR + / erastin - / RSL3 + |
|---|---|---|---|---|---|---|
| DMSO | 100 | 86 | 76 | 45 | 53 | 25 |
| Ferrostatin-1(5.0 μM) | 107 | 88 | 105 | 96 | 92 | 88 |
| Glutathione(1 mM) | 105 | 87 | 98 | 88 | 66 | 49 |
| Deferoxamine(50.0 μM) | 110 | 89 | 103 | 97 | 99 | 88 |
| Z-VAD-FMK(20.0 μM) | 107 | 87 | 77 | 48 | 57 | 28 |
| Necrostatin-1(20 μM) | 104 | 92 | 82 | 55 | 60 | 30 |
| 3-Methyladenine(1 mM) | 98 | 88 | 80 | 49 | 57 | 27 |

**G** MDA-MB-231 — Relative mRNA expression compared to Vehicle

| | Vehicle | SR | erastin | RSL3 | SR+erastin | SR+RSL3 |
|---|---|---|---|---|---|---|
| TGFβ | 1.00 | 0.79 | 0.58 | 0.87 | 0.34 | 0.35 |
| CCND1 | 1.00 | 0.70 | 0.61 | 0.63 | 0.58 | 0.27 |
| CCNE1 | 1.00 | 0.57 | 0.67 | 0.69 | 0.33 | 0.30 |
| CDC34 | 1.00 | 0.66 | 0.52 | 0.59 | 0.35 | 0.31 |
| CDK4 | 1.00 | 0.64 | 0.87 | 0.83 | 0.67 | 0.38 |
| CDKN1A | 1.00 | 1.42 | 1.90 | 1.72 | 3.58 | 4.16 |
| CDKN1B | 1.00 | 1.70 | 1.77 | 1.93 | 2.68 | 3.56 |
| CCNB1 | 1.00 | 0.96 | 0.95 | 1.16 | 0.97 | 0.86 |
| CCNF | 1.00 | 1.19 | 0.98 | 1.28 | 0.99 | 0.94 |
| CDKN3 | 1.00 | 0.74 | 0.94 | 0.93 | 0.86 | 0.78 |
| GADD45A | 1.00 | 1.71 | 1.80 | 2.08 | 2.91 | 3.42 |
| CCNA1 | 1.00 | 1.09 | 0.75 | 0.96 | 0.82 | 0.90 |
| TP53 | 1.00 | 0.93 | 0.88 | 0.75 | 0.38 | 0.23 |
| GTSE1 | 1.00 | 0.89 | 1.25 | 0.94 | 0.86 | 0.91 |
| MCM5 | 1.00 | 0.75 | 0.76 | 0.70 | 0.18 | 0.25 |
| MCM4 | 1.00 | 0.74 | 0.73 | 0.65 | 0.19 | 0.36 |
| CUL1 | 1.00 | 0.81 | 0.65 | 0.83 | 0.40 | 0.24 |
| MYC | 1.00 | 0.90 | 0.78 | 0.88 | 0.45 | 0.34 |
| MCM3 | 1.00 | 0.81 | 0.63 | 0.71 | 0.37 | 0.25 |
| MCM2 | 1.00 | 0.73 | 0.58 | 0.81 | 0.26 | 0.34 |

**G** HCC1937 — Relative mRNA expression compared to Vehicle

| | Vehicle | SR | erastin | RSL3 | SR+erastin | SR+RSL3 |
|---|---|---|---|---|---|---|
| TGFβ | 1.00 | 0.82 | 0.61 | 0.86 | 0.41 | 0.52 |
| CCND1 | 1.00 | 0.86 | 0.71 | 0.89 | 0.56 | 0.51 |
| CCNE1 | 1.00 | 0.79 | 0.74 | 0.74 | 0.49 | 0.49 |
| CDC34 | 1.00 | 0.74 | 0.61 | 0.80 | 0.27 | 0.21 |
| CDK4 | 1.00 | 0.82 | 0.69 | 0.58 | 0.70 | 0.59 |
| CDKN1A | 1.00 | 2.17 | 1.94 | 1.60 | 3.39 | 3.41 |
| CDKN1B | 1.00 | 1.72 | 1.90 | 1.94 | 2.39 | 2.75 |
| CCNB1 | 1.00 | 1.19 | 0.93 | 0.95 | 0.87 | 0.90 |
| CCNF | 1.00 | 1.20 | 0.99 | 0.96 | 0.97 | 0.94 |
| CDKN3 | 1.00 | 0.69 | 0.87 | 0.75 | 0.62 | 0.50 |
| GADD45A | 1.00 | 1.73 | 1.51 | 2.12 | 3.39 | 3.90 |
| CCNA1 | 1.00 | 0.86 | 1.19 | 0.98 | 0.95 | 0.88 |
| TP53 | 1.00 | 0.98 | 0.84 | 0.78 | 0.56 | 0.45 |
| GTSE1 | 1.00 | 0.84 | 0.90 | 0.95 | 0.93 | 0.89 |
| MCM5 | 1.00 | 0.71 | 0.75 | 0.68 | 0.40 | 0.50 |
| MCM4 | 1.00 | 0.82 | 0.87 | 0.68 | 0.29 | 0.79 |
| CUL1 | 1.00 | 0.85 | 0.92 | 0.77 | 0.38 | 0.51 |
| MYC | 1.00 | 0.89 | 0.79 | 0.65 | 0.45 | 0.42 |
| MCM3 | 1.00 | 0.81 | 0.75 | 0.87 | 0.59 | 0.42 |
| MCM2 | 1.00 | 0.89 | 0.75 | 0.75 | 0.48 | 0.40 |

# Supplementary Figure 5

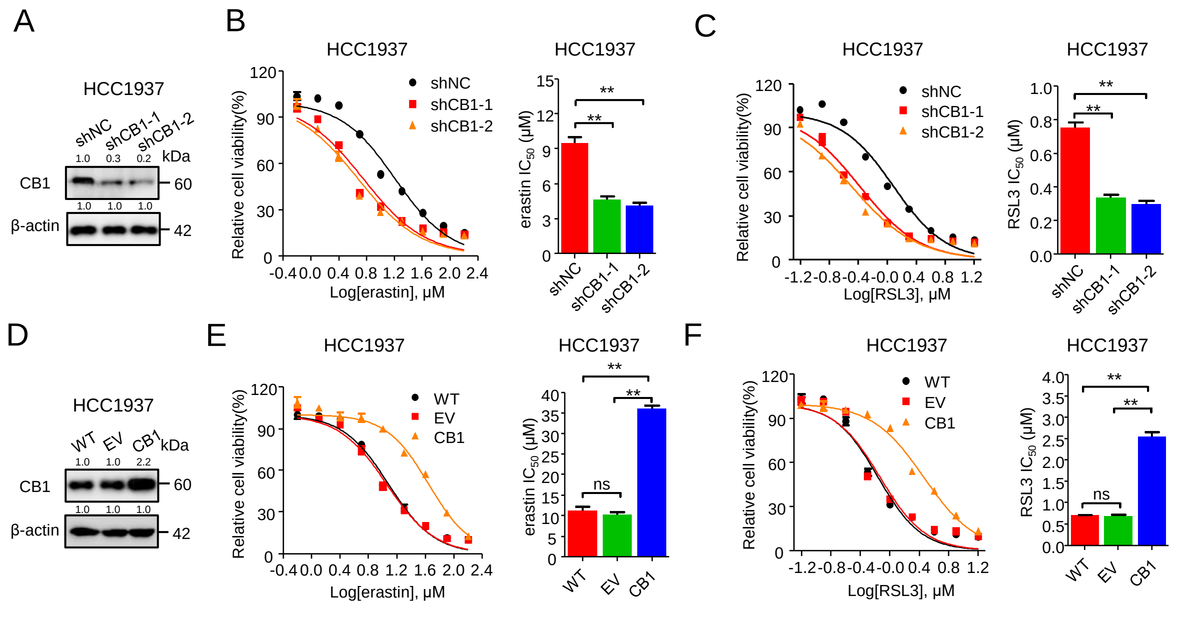

# Supplementary Figure 6

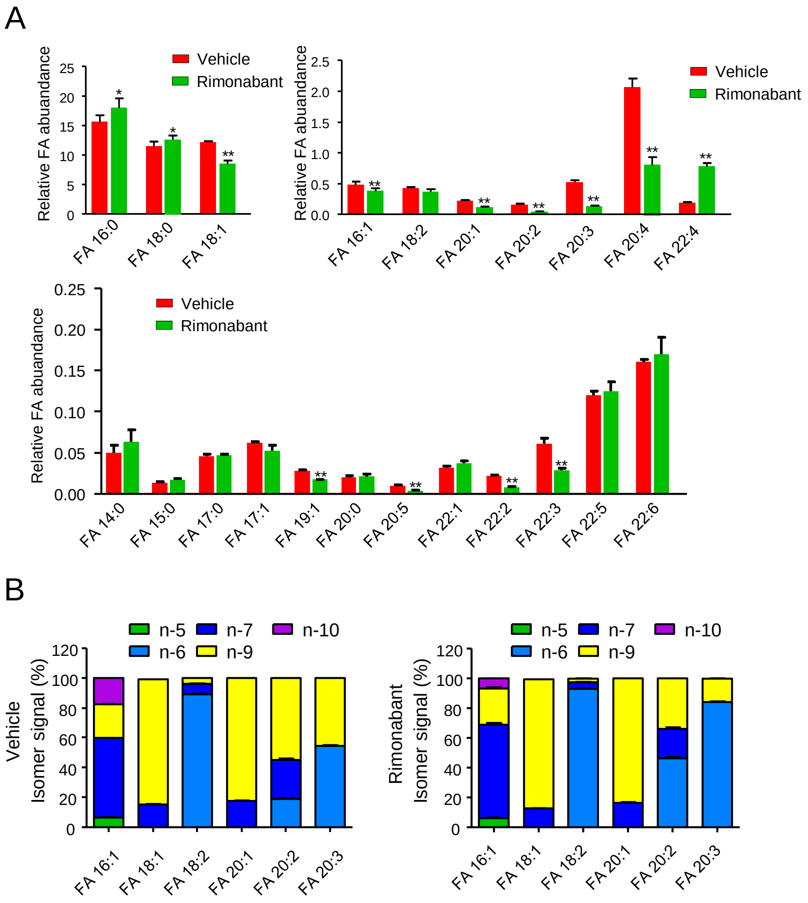

Supplementary Figure 7

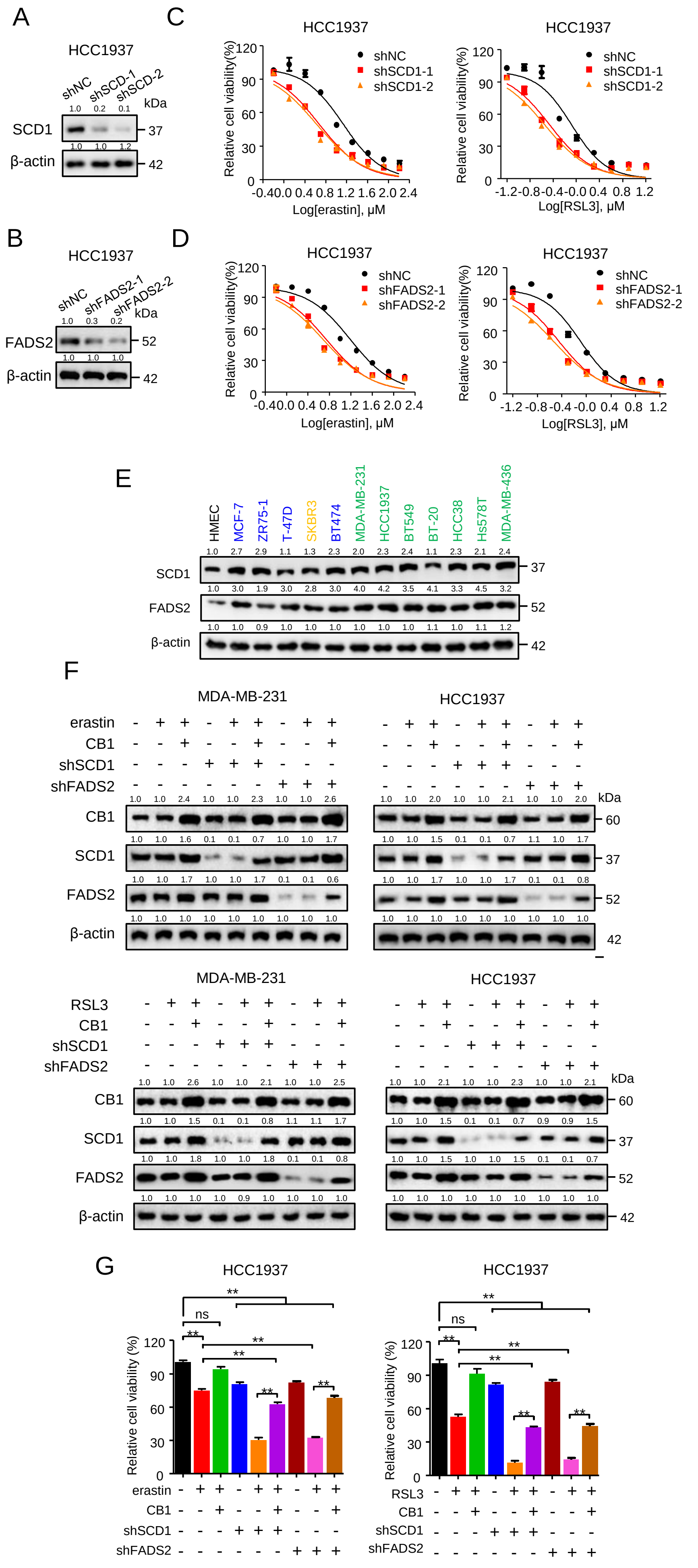

Supplementary Figure 8

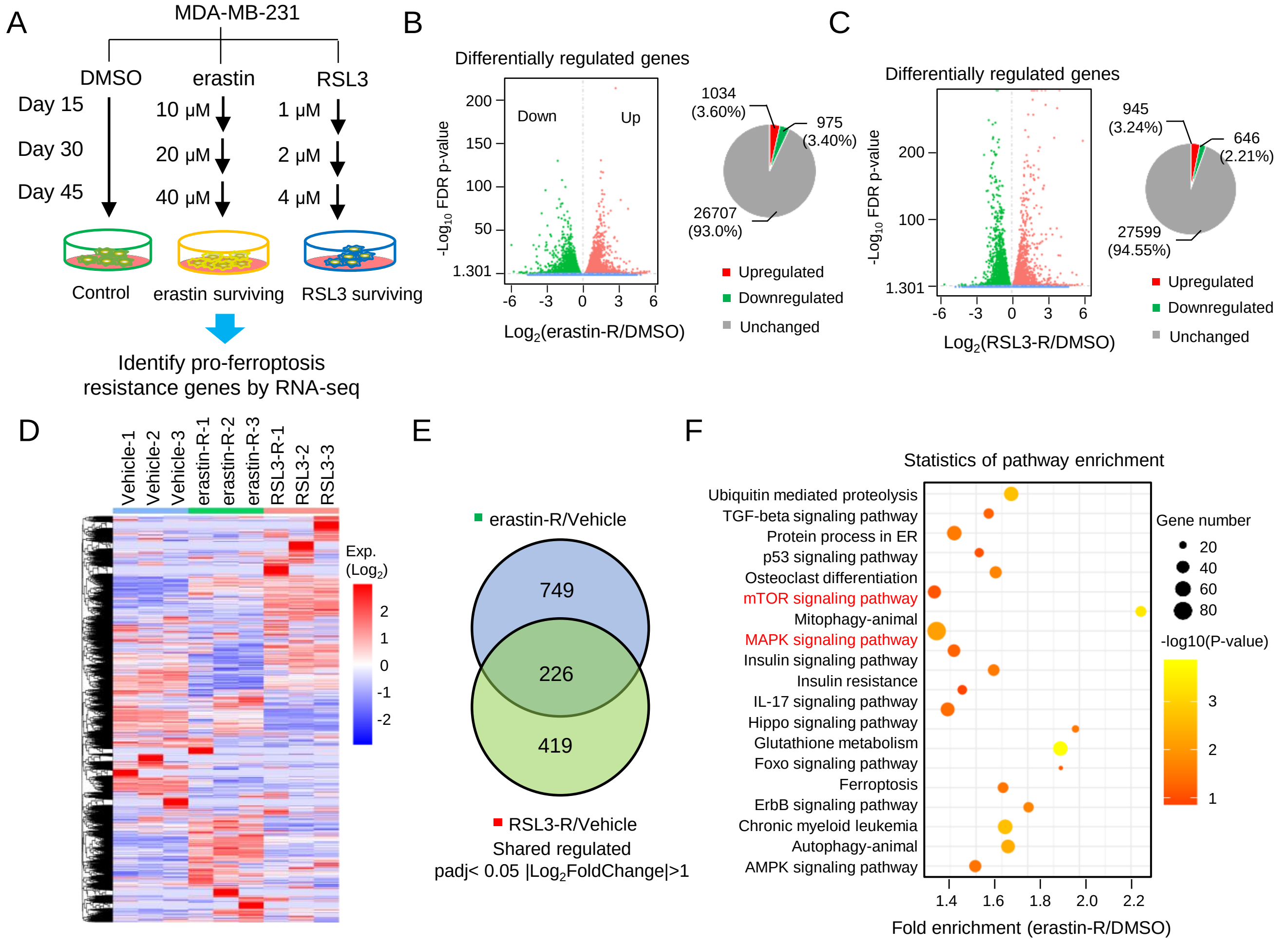

# Supplementary Figure 9

A

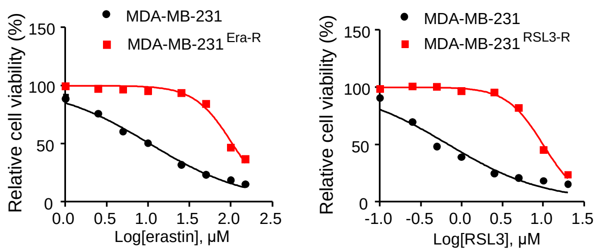


| Cell line | erastin $IC_{50}$ (μM) |
|---|---|
| MDA-MB-231 | 9.55±0.66 |
| MDA-MB-231$^{Era-R}$ | 114.4±6.34 |

| Cell line | RSL3 $IC_{50}$ (μM) |
|---|---|
| MDA-MB-231 | 0.71±0.11 |
| MDA-MB-231$^{RSL3-R}$ | 9.93±0.61 |

B

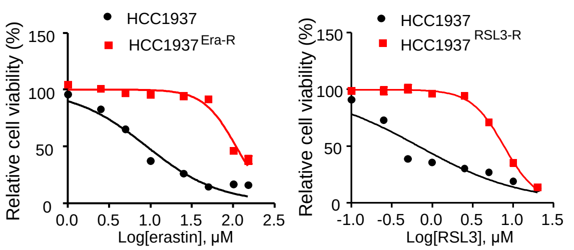


| Cell line | erastin $IC_{50}$ (μM) |
|---|---|
| HCC1937 | 11.58±0.98 |
| HCC1937$^{Era-R}$ | 101.4±2.74 |

| Cell line | RSL3 $IC_{50}$ (μM) |
|---|---|
| HCC1937 | 0.85±0.07 |
| HCC1937$^{RSL3-R}$ | 7.72±0.83 |

C

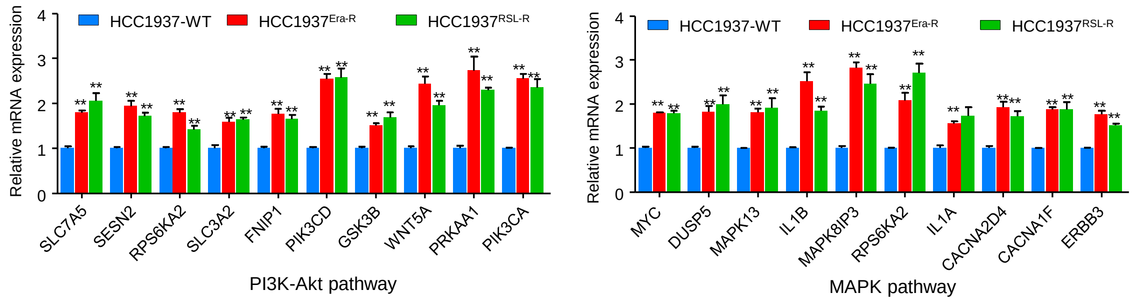


D

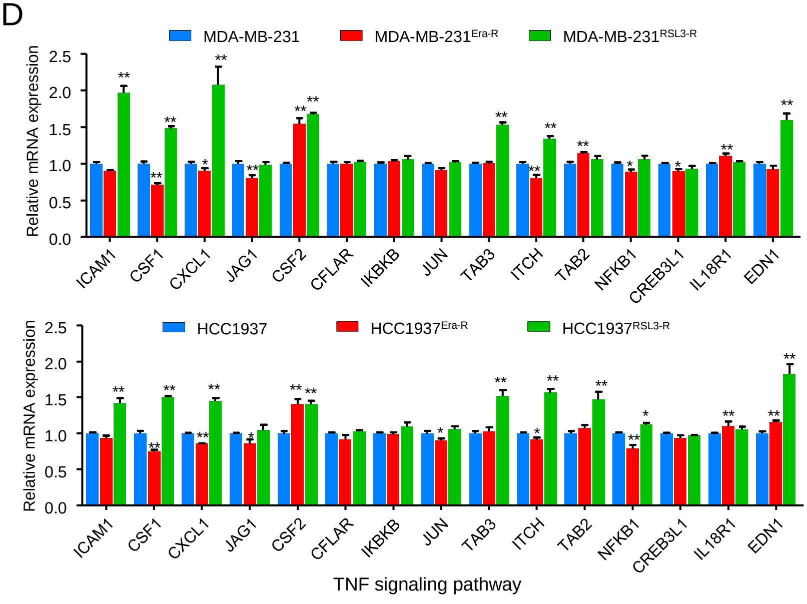

Supplementary Figure 10

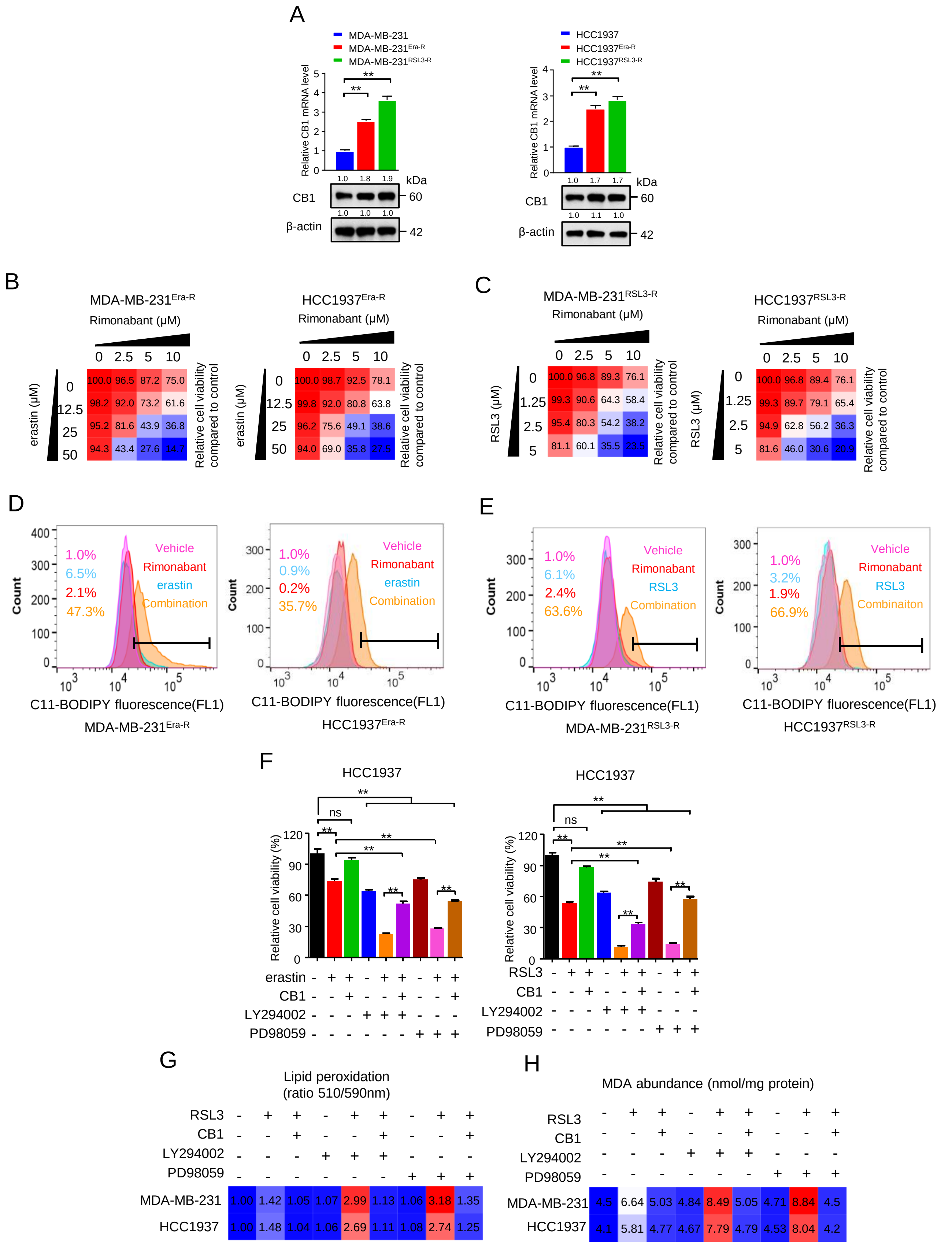

# Supplementary Figure 11

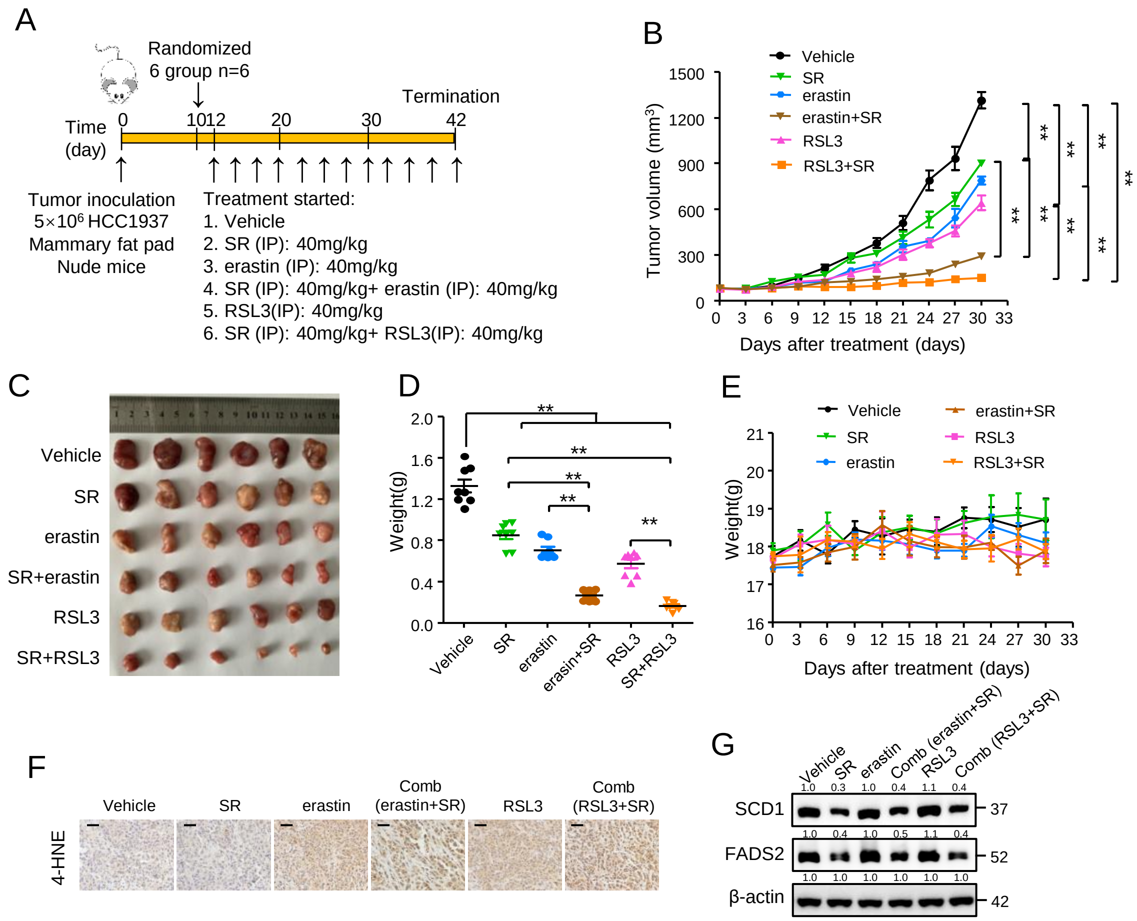

# Supplementary Figure 12

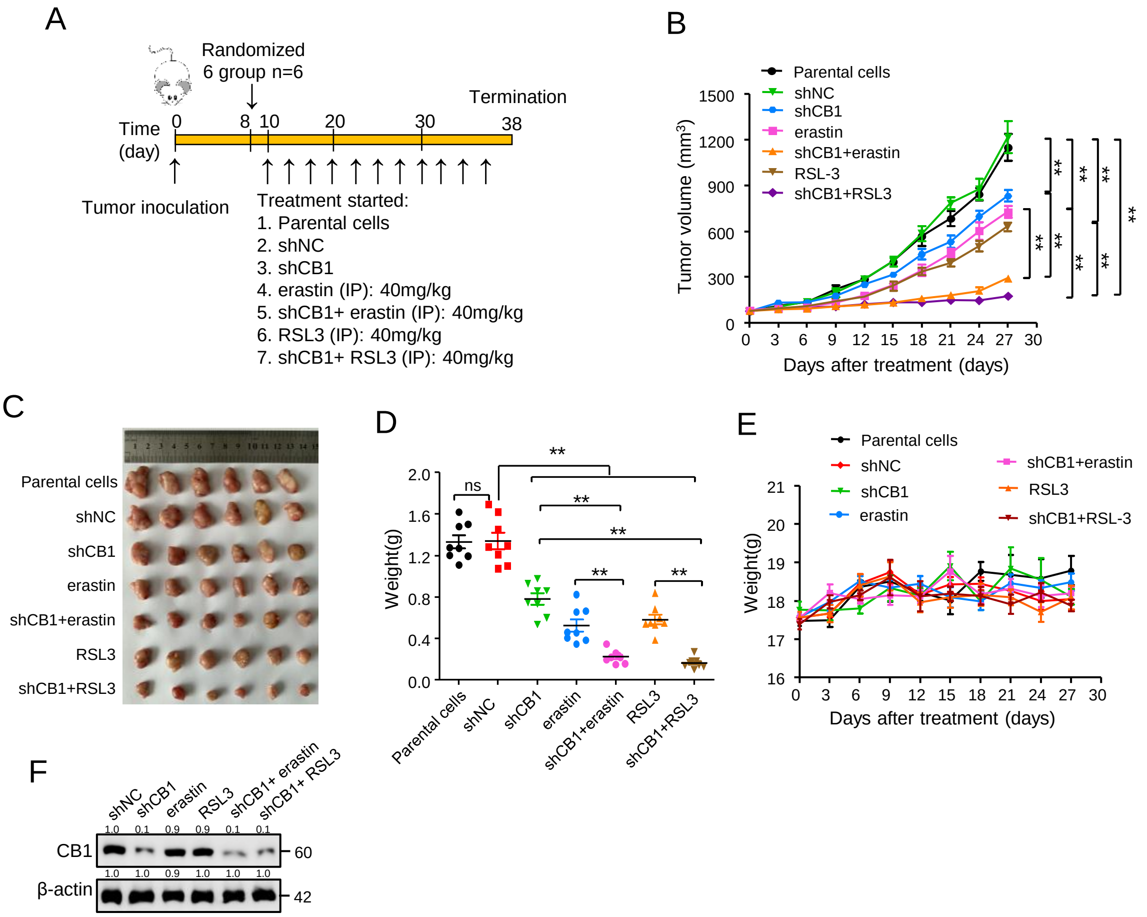

Supplement: Supplementary file 1 — Supplementary Figures [file 41419_2022_5242_MOESM1_ESM.pdf]
