# Supplementary material for: Inhibition of cannabinoid receptor type 1 sensitizes triple-negative breast cancer cells to ferroptosis via regulating fatty acid metabolism
Source: Cell Death Dis. 2022 Sep 21;13(9):808. doi: 10.1038/s41419-022-05242-5 (PMC9492666; doi:10.1038/s41419-022-05242-5)
Supplement: Supplementary file 2 — Supplementary Figure legends [file 41419_2022_5242_MOESM2_ESM.docx]

**Supplementary Figure legends**

**S-Figure 1 Synergy of CB1 antagonists with ferroptosis inducers in inhibiting growth of TNBC cells**.

**(A)** Volcano plot showing the results of drug screen in HCC1937 cells. Synergy Index_(erastin)_ and Synergy Index_(RSL3)_ representing the combinational strength of 303 lipid metabolism compounds with erastin and RSL3, respectively for 72 h. Significant combinational treatment of CB1 antagonists with erastin and RSL3, respectively, are shown in red. **(B)** Combinational effects of CB1 antagonists (10 μM) with ferroptosis inducers (erastin, RSL3, ML210, FIN56, CIL56) at the indicated concentration in HCC1937 cells treated with the inhibitors of single agent or the indicated target pairs. Viability was measured 72h after treatment with the indicated concentrations of drugs. Effects on cell viability were calculated as percentage of vehicle treated cells. **(C)** Dose-response matrix for rimonabant (SR) and erastin combination in MDA-MB-231 (left) and HCC1937 (right) cells. Cell viability was measured 72 h after treatment with the indicated concentrations of drugs. **(D)** Dose-response matrix for rimonabant (SR) and RSL3 combination in MDA-MB-231 (left) and HCC1937 (right) cells. Cell viability was measured 72 h after treatment with the indicated concentrations of drugs. **(E)** Effects of erastin and rimonabant (SR) as single agents or drug combinations in HCC1937 cells. The cell viability was assessed by CCK-8 assay after treatment for 72 h with the indicated doses of the drugs. **(F)** Effects of RSL3 and rimonabant (SR) as single agents or drug combinations in HCC1937 cells. The cell viability was assessed by CCK-8 assay after treatment for 72h with the indicated doses of the drugs. Data shown are mean ± SD of triplicate measurements that were repeated 3 times with similar results.

**S-Figure 2 No synergy was observed when rimonabant combined with Doxorubicin, Taxol, Carboplatin, Olaparib on cell viability.**

**(A-D)** Dose-response curves of rimonabant (SR) of doxorubicin **(A)**, Taxol **(B)**, carboplatin **(C)**, olaparib **(D)** as single agent or drug combination in MDA-MB-231 and HCC1937 cells. Cell viability was measured 72 h after treatment with the indicated doses of the drugs (left). Combinational Index (CI) was calculated by the Chou-Talalay equation using multiple doses and response points. CI values for three different indicated Fa are shown (right). Data shown are mean ± SD of triplicate measurements that were repeated 3 times with similar results.

**S-Figure 3 Synergy of rimonabant with ferroptosis inducers in decreasing growth of TNBC cells**.

**(A-B)** Dose-response curves erastin **(A)** or RSL3 **(B)** as single agents or combined with rimonabant (SR) at the indicated doses in the HCC1937 cells for 72 h. The effects of SR on the IC_50_ of erastin or RSL3 are shown in the bar graphs (right). **(C)** The proliferation curve of single agents and drug combinations in the HCC1937 cells treated with indicated concentrations of erastin and SR (left) or RSL3 (right) and rimonabant (SR) for 96 h. The cell viability was assessed by CCK-8 assay treated with the indicated doses of the drugs. **p < 0.01 (one-way ANOVA). **(D)** In colony formation assays, HCC1937 cells were treated with rimonabant (SR, 10 μM) or vehicle combined with the increasing concentrations of erastin (left) or RSL3 (right) for 14 days. Data shown are mean ± SD of triplicate measurements that were repeated 3 times with similar results.

**S-Figure 4 Rimonabant combined with erastin or RSL3 significantly inhibited TNBC cell growth by inducing ferroptosis.**

**(A)** HCC1937 cells were pretreated with the indicated cell death inhibitors for 1 hour, and then cells were added with rimonabant (SR, 10 μM), erastin (5 μM) and RSL3 (0.5 μM) as single agents or drug combinations for an additional 72 hours. Cell viability (CCK-8) is presented as percentage of untreated cells. **(B)** The level of GSH was determined after treated with rimonabant (SR, 10 μM), erastin (5 μM) and RSL3 (0.5 μM) as single agents or drug combinations for 48 h in HCC1937 cells. ns (no significance), **p < 0.01 (one-way ANOVA). **(C-F)** MDA (**C**), 4-HNE (**D**), lipid peroxidation (**E**) and cytosolic ROS (**F**) production of HCC1937 cells treated as **(B)** Histograms show the production of MDA, 4-HEN, relative fold change of lipid ROS, and cytosolic ROS (right panel). ns (no significance), **p < 0.01 (one-way ANOVA). **(G)** qPCR analysis of indicated gene expression associated with cell cycle regulation in MDA-MB-231 (left) and HCC1937 (right) cells treated as **(B)**. Data shown are mean ± SD of triplicate measurements that were repeated 3 times with similar results.

**S-Figure 5 CB1 modulated the sensitivity of TNBC cells to ferroptosis.**

**(A-C)** Effect of CB1 knockdown on HCC1937 cells in response to erastin/RSL3 treatment with two distinct target gene shRNA expression vectors. WB analysis was conducted to detect CB1 expression in HCC1937 cells that were stably transfected with negative control shRNA (shNC) or shCB1 **(A)**. Dose-response curves of HCC1937 cell stably transfected with n negative control shRNA (shNC) or shCB1 were treated with erastin **(B)** or RSL3 **(C)** at the indicated doses. Cell viability was assessed by CCK-8 assay. The effects of negative control vector or shCB1 on the IC_50_ of erastin or RSL3 are shown in the bar graphs (right). **p < 0.01 (*t* test). **(D-F)** Effects of CB1 overexpression on HCC1937 cells in response to erastin and RSL3 treatment, respectively. WB analysis was conducted to detect CB1 expression in parental HCC1937 cells and cells that were stably transfected with empty vector or CB1 **(D)**. Dose-response curves of HCC1937 cell viability after stably transfected with empty vector or CB1 treated with erastin **(E)** or RSL3 **(F)** at the indicated doses. Cell viability was assessed by CCK-8 assay. The effects of empty vector, CB1 overexpression on the IC_50_ of erastin or RSL3 are shown in the bar graphs (right). ns (no significance), **p < 0.01 (one-way ANOVA). Data shown are mean ± SD of triplicate measurements that were repeated 3 times with similar results.

**S-Figure 6 Fatty acid profiles caused by rimonabant.**

**(A)** Relative fatty acid abundance of rimonabant treated MDA-MB-231 cells showing change in mono- and poly-unsaturated fatty acid isomers from the hydrolyzed lipid pool. **(B)** Fractional distribution profiles for 6 mono- and polyunsaturated fatty acids from the hydrolyzed lipid pool (n = 3; mean fractional distribution displayed), FA (fatty acid). Data shown are mean ± SD of triplicate measurements that were repeated 3 times with similar results. Statistical significance was assessed by two-tailed Student’s t test. *p < 0.05, **p < 0.01 versus the corresponding control.

**S-Figure 7 SCD1 and FADS2 knockdown increased sensitivity of TNBC cells to ferroptosis.**

**(A-D)** Effect of SCD1 and FADS2 knockdown on HCC1937 cells in response to erastin and RSL3 treatment, respectively, using two distinct target gene shRNA expression vectors. WB analysis was conducted to detect SCD1 **(A)** and FADS2 **(B)** in HCC1937 cells that were stably transfected with negative control shRNA (shNC), shSCD1 or shFADS2. Dose-response curves of HCC1937 cell stably transfected with negative shRNA (shNC), shSCD1 **(C)** or shFADS2 **(D)** treated with erastin (left) or RSL3 (right) at the indicated doses. The cell viability was assessed by CCK-8 assay after treatment for 72 h with the indicated doses of the drugs. **(E)** WB analysis of SCD1 and FADS2 in the indicated breast cell lines, including normal-like cells (black), 4 luminal cell lines (blue), HER2-enriched cell line (orange) and 7 TNBC cell lines (green). **(F)** WB analysis of MDA-MB-231 and HCC1937 cells transfected with empty vector, CB1, shSCD1 or shFADS2 treated with erastin (up) or RSL3 (down). **(G)** The cell viability of HCC1937 cells stably transfected with empty vector, CB1, shSCD1 or shFADS2 treated with erastin (5 μM) or RSL3 (0.5 μM). The cell viability was assessed measured 72h after treatment with the indicated doses of the drugs. ns (no significance), **p < 0.01 (one-way ANOVA). Data shown are mean ± SD of triplicate measurements that were repeated 3 times with similar results.

**S-Figure 8 RNA-seq of MDA-MB-231 cells surviving from increased concentration treatment of erastin/RSL3.**

**(A)** Treatment schedule for the surviving MDA-MB-231 cells treated with increased amount of erastin or RSL3 to perform RNA sequencing (RNA-seq). **(B-C)** A volcano plot illustrating differentially regulated gene expression from RNA-seq analysis of MDA-MB-231 cells between the control vehicle with surviving from increased erastin treatment (erastin-R) **(B)** or RSL3 treatment (RSL3-R) **(C)**. Genes upregulated and downregulated are shown in red and green, respectively. Values are presented as the log_10_ of tag counts. **(D)** Heat map of transcriptional profile denoting unbiased clustering of MDA-MB-231 cells treated with erastin, RSL3 or vehicle control (DMSO) (n=3). Total RNA was analyzed by high-throughput whole transcriptome sequencing (RNA-seq). Significant differential expression defined as an absolute log_2_ (fold change) ≥1 and padj < 0.05. **(E)** Overlay plots of regulated genes of MDA-MB-231 cells transcriptome surviving from increased erastin treatment (erastin-R) or RSL3 treatment (RSL3-R). **(F)** KEGG pathway analysis of differentially expressed genes in MDA-MB-231 cells transcriptome surviving from increased erastin treatment (erastin-R) (The top 20 most significantly activated pathway are shown).

**S-Figure 9 PI3K-AKT and MAPK pathways were hyperactivated in erastin-/RSL3-resistant TNBC cells.**

**(A)** Dose-response curves of control MDA-MB-231 and erastin resistant MDA-MB-231 cells (MDA-MB-231^Era-R^) treated with the indicated concentrations of erastin (left). Dose-response curves of control MDA-MB-231 and RSL3 resistant MDA-MB-231 cells (MDA-MB-231^RSL3-R^) treated with the indicated concentrations of RSL3 (right). The cell viability was assessed by CCK-8 assay after treatment for 72 h with the indicated doses of the drugs. **(B)** Dose-response curves of control HCC1937 and erastin resistant HCC1937 cells (HCC1937^Era-R^) treated with the indicated concentrations of erastin (left). Dose-response curves of control HCC1937 and RSL3 resistant HCC1937 cells (HCC1937^RSL3-R^) treated with the indicated concentrations of RSL3 (right). The cell viability was assessed by CCK-8 assay after treatment for 72 h with the indicated doses of the drugs. **(C)** qPCR analysis of the indicated gene expression associated with PI3K-AKT pathway (left) and MAPK pathway (right) in HCC1937^Era-R^ and HCC1937^RSL3-R^ cells, respectively, compared with parental HCC1937 cells. *p < 0.05, **p < 0.01 (*t* test). **(D)** qPCR analysis of the indicated gene expression associated with TNF signaling pathway MDA-MB-231, MDA-MB-231^Era-R^, MDA-MB-231^RSL3-R^ and HCC1937 cells, HCC1937^Era-R^, HCC1937^RSL3-R^ cells, respectively. *p < 0.05, **p < 0.01 (*t* test). Data shown are mean ± SD of triplicate measurements that were repeated 3 times with similar results.

**S-Figure 10 PI3K-AKT and MAPK pathways were involved in CB1 repressed the ferroptosis sensitivity.**

**(A)** qPCR and WB analysis of relative CB1 expression in MDA-MB-231, MDA-MB-231^Era-R^, MDA-MB-231^RSL3-R^ and HCC1937, HCC1937^Era-R^, HCC1937^RSL3-R^ cells. **p < 0.01 (*t* test) compared with parental cells. **(B)** Effect of rimonabant and erastin as single agent or combination treatment on reducing cell viability in erastin-resistant MDA-MB-231 (MDA-MB-231^Era-R^) and HCC1937 (HCC1937^Era-R^) cells. **(C)** Effect of rimonabant and RSL3 as single agent or combination treatment on reducing cell viability in RSL3-resistant MDA-MB-231 (MDA-MB-231^RSL3-R^) and HCC1937 (HCC1937^RSL3-R^) cells. **(D)** Effect of rimonabant and erastin as single agent or combination treatment on lipid peroxidation in erastin-resistant MDA-MB-231 (MDA-MB-231^Era-R^) and HCC1937 (HCC1937^Era-R^) cells. **(E)** Effect of rimonabant and RSL3 as single agent or combination treatment on lipid peroxidation in RSL3-resistant MDA-MB-231 (MDA-MB-231^RSL3-R^) and HCC1937 (HCC1937^RSL3-R^) cells. **(F)** The cell viability of HCC1937 cells stably transfected with empty vector or CB1 treated with LY294002 (5 μM) or PD98059 (10 μM). The cell viability was assessed by CCK-8 assay after treatment for 72 h with the indicated doses of the drugs. ns (no significance), **p < 0.01 (one-way ANOVA). **(G-H)** Effect of CB1 overexpression, LY294002 (5 μM) and PD98059 (10 μM) on lipid peroxidation **(G)** and MDA **(H)** response to RSL3 treatment, respectively, in MDA-MB-231 and HCC1937 cells. Data shown are mean ± SD of triplicate measurements that were repeated 3 times with similar results.

**S-Figure 11 Rimonabant combined with erastin/RSL3 significantly repressed TNBC tumor growth *in vivo*.**

**(A)** Treatment schedule for the HCC1937 cells orthotopic implantation model was treated with vehicle, rimonabant (SR), erastin and RSL3 as single agents or drug combinations. **(B-D)** Mice were treated as **(A)** 12 days after xenograft, and tumor size was monitored every other day **(B)**. Photograph of tumors **(C)** and tumor weight **(D)** were shown (n = 6 per group). **p < 0.01 (one-way ANOVA). **(E)** Body weight of BALB/c nude mice treated as **(A)** were shown (n = 6 per group). **(F)** Representative images of 4-HNE IHC staining in harvested tumors from each group were shown. Scale bars represent 50 mm. **(G)** WB analysis of SCD1 and FADS2 in harvested tumors from each group were shown. Data shown are the means ± SD from six tumors at each time point.

**S-Figure 12 CB1 knockdown significantly enhanced the effect of erastin/RSL3 on reducing TNBC tumor growth *in vivo*.**

**(A)** Treatment schedule for the CB1 shRNA lentivirus or negative control shRNA (shNC), or parental MDA-MB-231 cells orthotopic implantation model was treated with vehicle, erastin or RSL3. **(B-D)** Mice were treated as **(A)** 10 days after xenograft, and tumor size was monitored every other day **(B)**. Photograph of tumors **(C)** and tumor weight **(D)** were shown (n = 6 per group). ns (no significance), **p < 0.01 (one-way ANOVA). **(E)** Body weight of BALB/c nude mice treated as **(A)** were shown (n = 6 per group). **(F)** WB analysis of CB1 in harvested tumors from each group were shown. Data shown are the means ± SD from six tumors at each time point.
