# Supplementary material for: Inhibition of cannabinoid receptor type 1 sensitizes triple-negative breast cancer cells to ferroptosis via regulating fatty acid metabolism
Source: Cell Death Dis. 2022 Sep 21;13(9):808. doi: 10.1038/s41419-022-05242-5 (PMC9492666; doi:10.1038/s41419-022-05242-5)
Supplement: Supplementary file 7 — Original full length WB [file 41419_2022_5242_MOESM7_ESM.pdf]

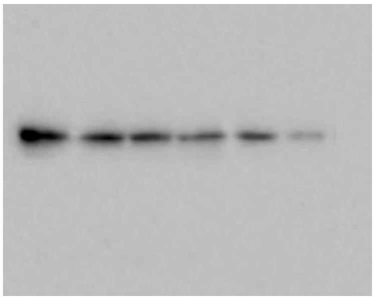

Figure 2-C-HCC1937-cyclinD1

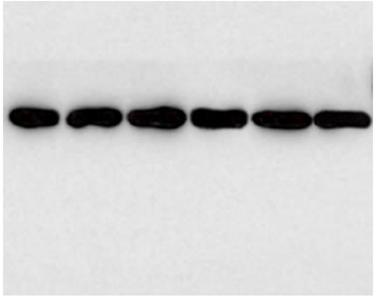

Figure 2-C-MDA-MB-231-CYCLINB1

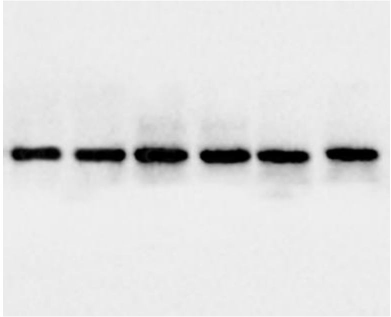

Figure 2-C-HCC1937-cyclinB1

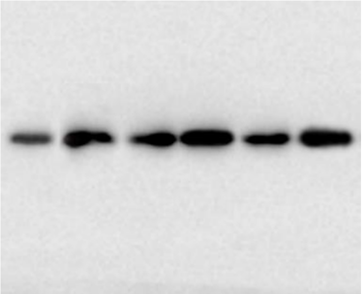

Figure 2-C-MDA-MB-231-P21

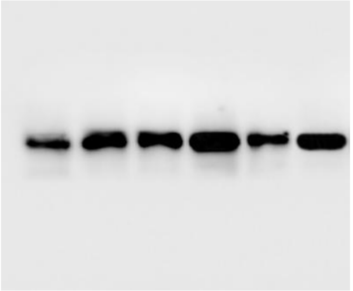

Figure 2-C-HCC1937-p21

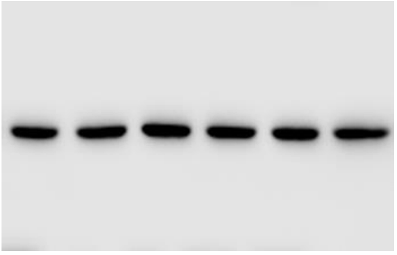

Figure 2-C-MDA-MB-231-actin

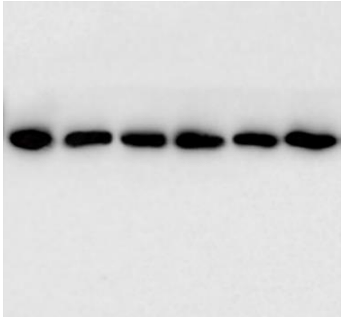

Figure 2-C-HCC1937-actin

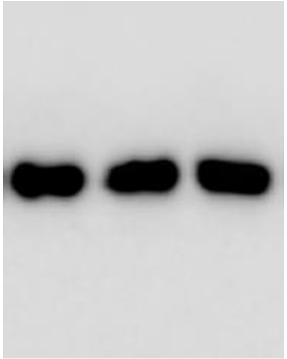

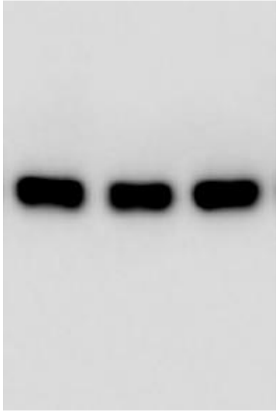

Figure 3-A-MDA-MB-231-actin

Figure 3-D-MDA-MB-231-actin

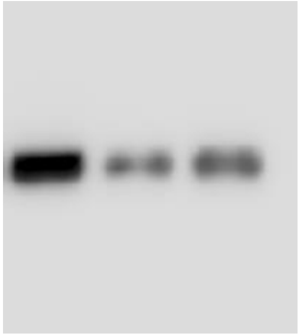

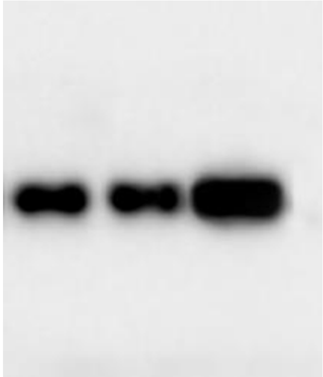

Figure 3-A-MDA-MB-231-CB1

Figure 3D-MDA-MB-231-CB1

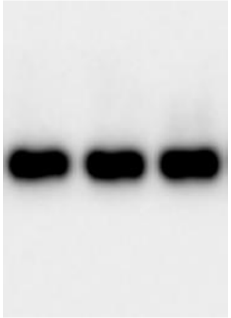

FIGURE 5D- HCC1937 -actin

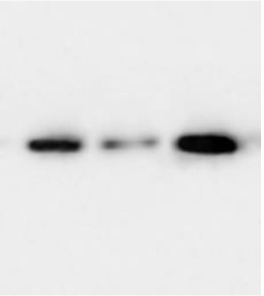

FIGURE 5D-HCC1937-CB1

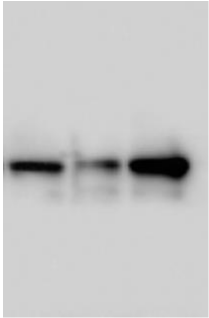

FIGURE 5D-HCC1937-FADS2

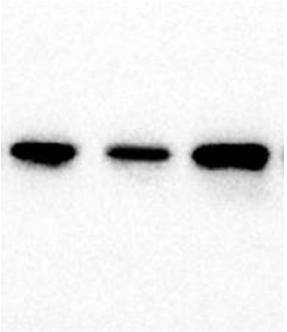

FIGURE 5D-HCC1937-SCD1

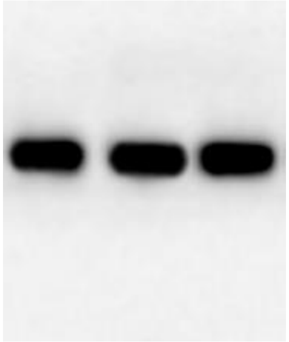

FIGURE 5D-MDA-MB-231-actin

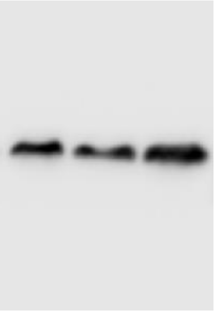

FIGURE 5D-MDA-MB-231-CB1

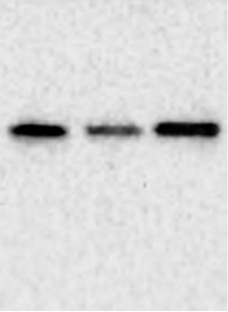

FIGURE 5D-MDA-MB-231-FADS2

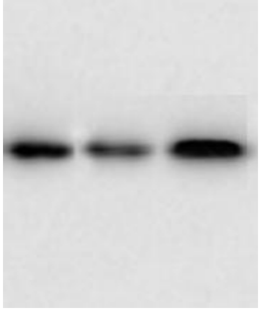

FIGURE 5D-MDA-MB-231-SCD1

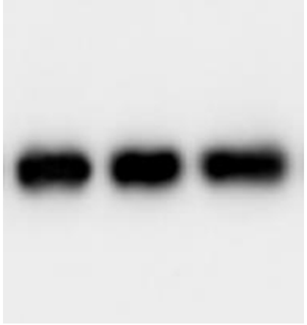

FIGURE 5E-MDA-MB-231-actin

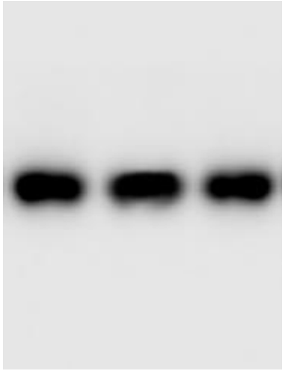

FIGURE 5F-MDA-MB-231-actin

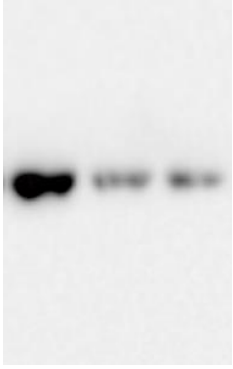

FIGURE 5-F-MDA-MB-231-FADS2

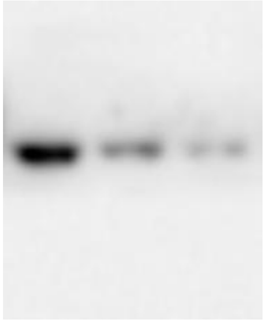

FIGURE 5-F-MDA-MB-231-SCD1

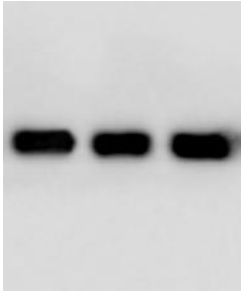

FIGURE 5-C-HCC1937-ACTIN

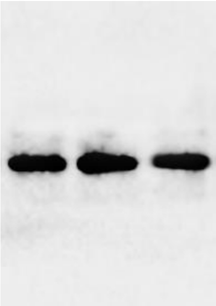

FIGURE 5-C-HCC1937-AKT

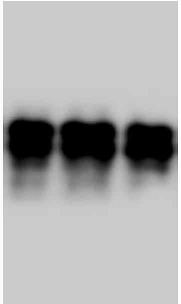

FIGURE 5-C-HCC1937-ERK

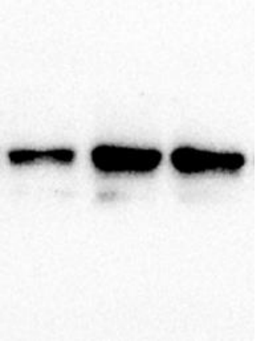

FIGURE 5-C-HCC1937-PAKT

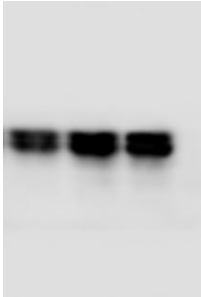

FIGURE 5-C-HCC1937-PERK

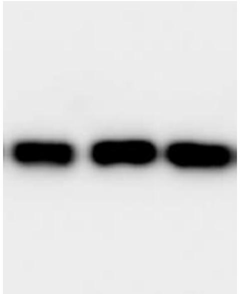

FIGURE 5-C-MDA-MB-231-ACTIN

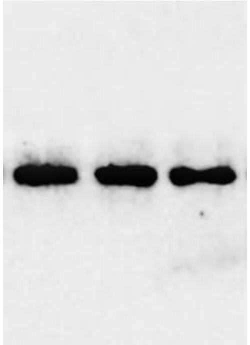

FIGURE 5-C-MDA-MB-231-AKT

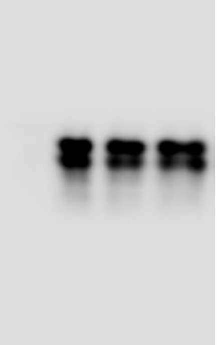

FIGURE 5-C-MDA-MB-231-ERK

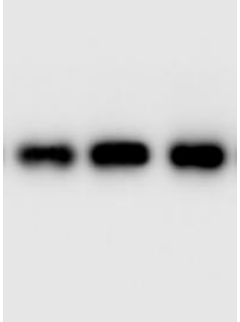

FIGURE 5-C-MDA-MB-231-PAKT

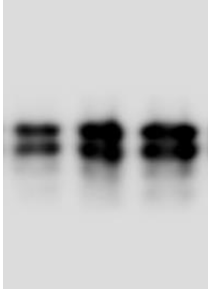

FIGURE 5-C-MDA-MB-231-PERK

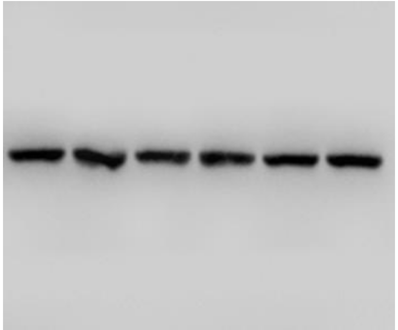

Figure 5F-HCC1937-actin

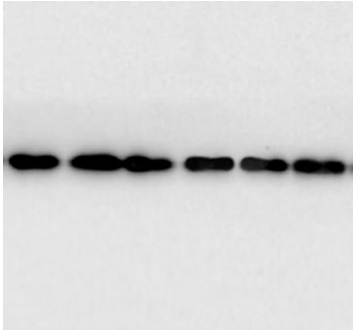

Figure 5F-HCC1937-AKT

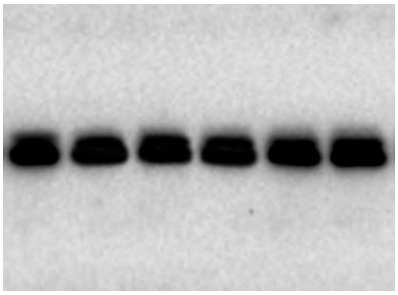

Figure 5F-HCC1937-ERK

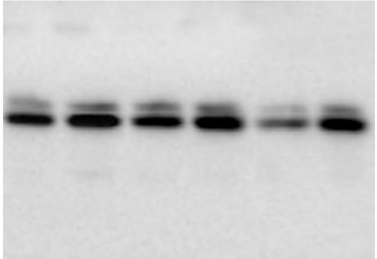

Figure 5F-HCC1937-pERK

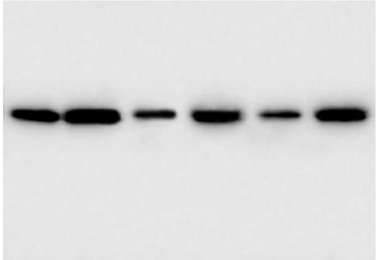

Figure 5F-HCC1937-SCD1

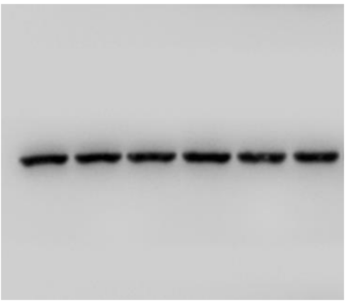

Figure 5F-MDA-MB-231-actin

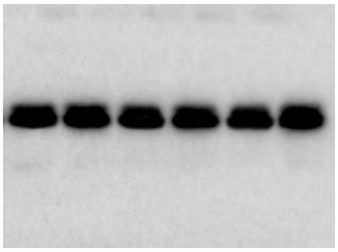

Figure 5F-MDA-MB-231-ERK

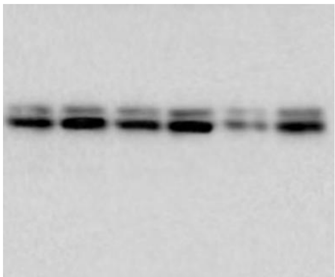

Figure 5F-MDA-MB-231-pERK

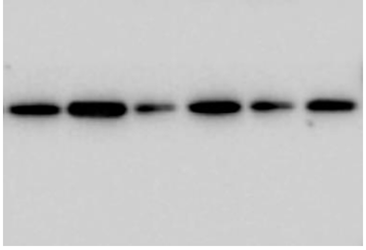

Figure 5F-MDA-MB-231-SCD1

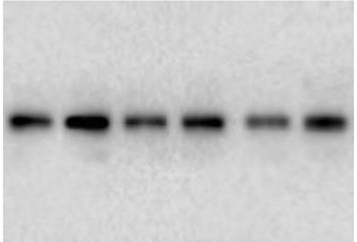

Figure-5F-HCC1937-FADS2

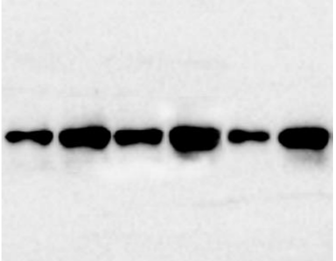

Figure-5F-HCC1937-CB1

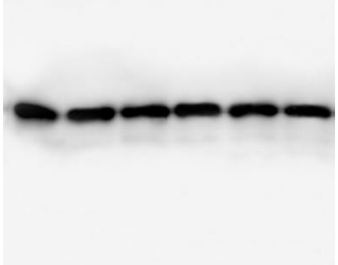

Figure-5F-MDA-MB-231-AKT

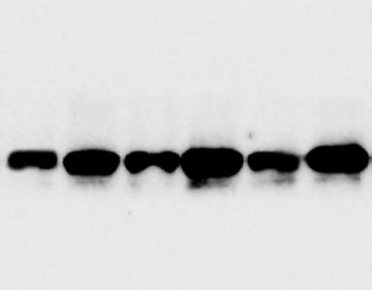

Figure-5F-MDA-MB-231-CB1

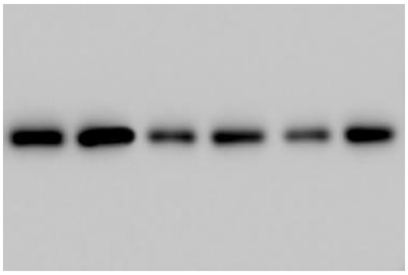

Figure-5F-MDA-MB-231-FADS2

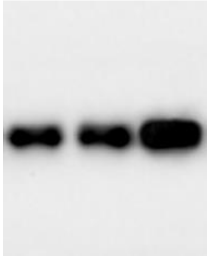

S-Figure 5-D-HCC1937-CB1

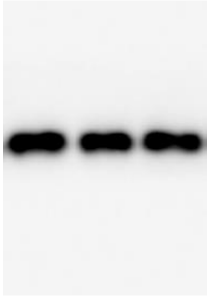

S-Figure 5-A-HCC1937-actin

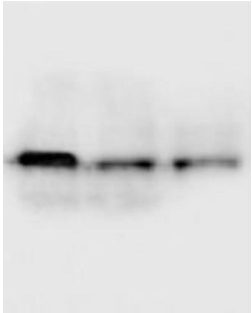

S-Figure 5-A-HCC1937-CB1

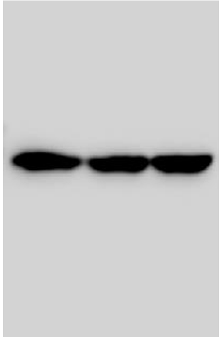

S-Figure 5D- HCC1937 -actin

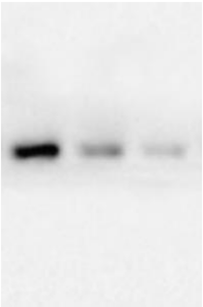

S-Figure 7A-HCC1937-SCD1

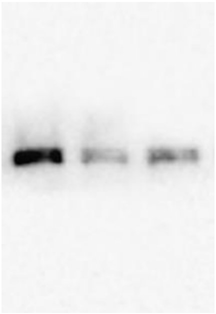

S-Figure 7-B-HCC1937-FADS2

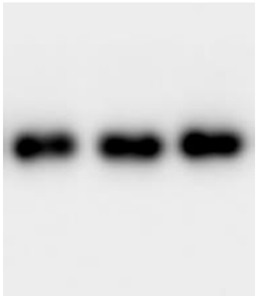

S-Figure 7A-HCC1937-actin

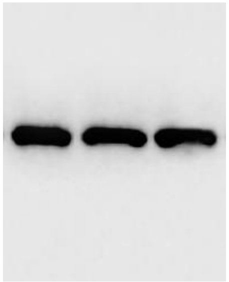

S-Figure 7-B-HCC1937-actin

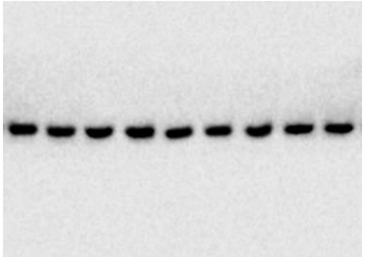

S-Figure-7E-actin-MDA-MB-231-B

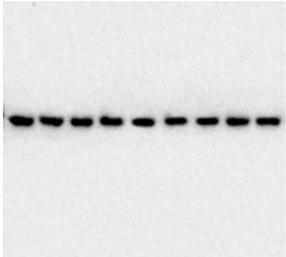

S-Figure-7E-actin-HCC1937-B

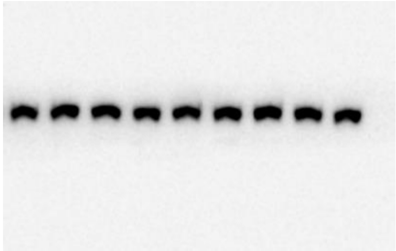

S-Figure-7E-actin-MDA-MB-231-A

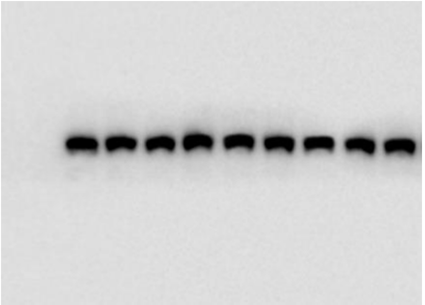

S-Figure-7E-actin- HCC1937-B

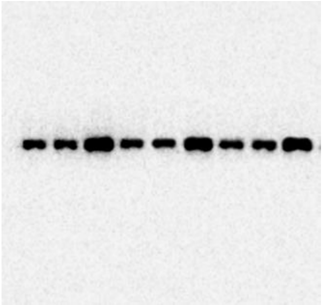

S-Figure-7E-CB1-MDA-MB-231-B

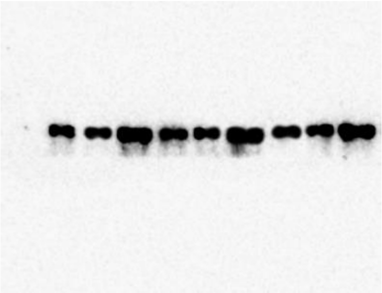

S-Figure-7E-CB1-HCC1937-B

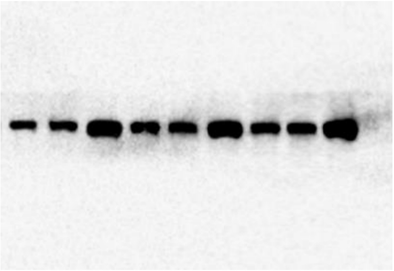

S-Figure-7E-CB1-MDA-MB-231-A

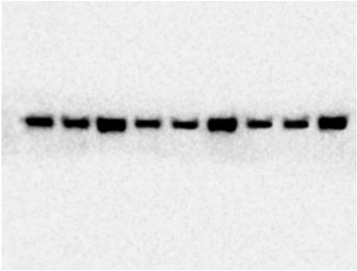

S-Figure-7E-CB1-HCC1937-B

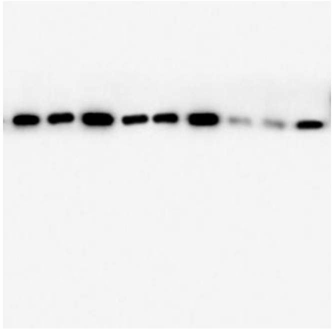

S-Figure-7E-FADS2-HCC1937-B

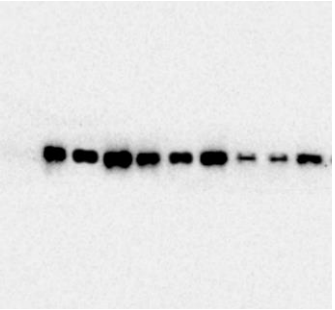

S-Figure-7E-FADS2-HCC1937-B

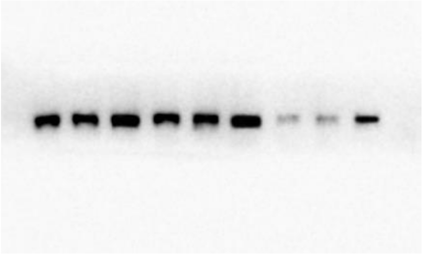

S-Figure-7E-FADS2-MDA-MB-231-A

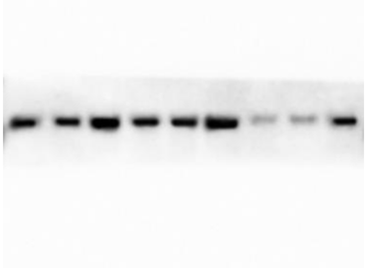

S-Figure-7E-FADS2- HCC1937-A

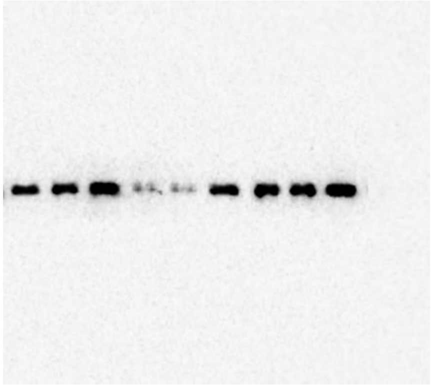

S-Figure-7E-SCD1-MDA-MB-231-B

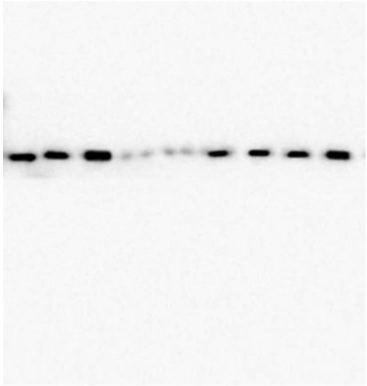

S-Figure-7E-SCD1-HCC1937-B

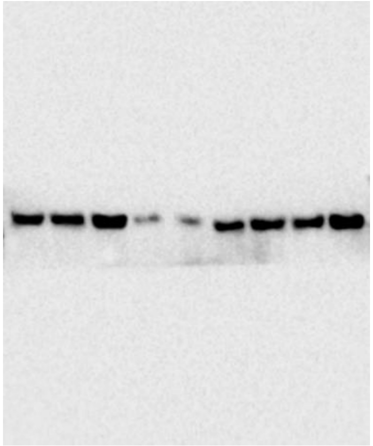

S-Figure-7E-SCD1-MDA-MB-231-A

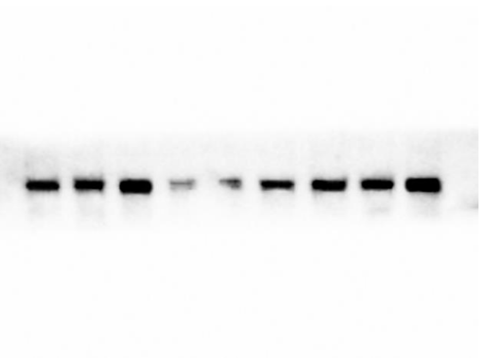

S-Figure-7E-SCD1- HCC1937-A

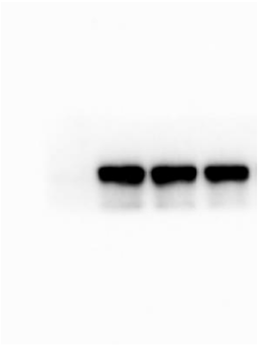

S-Figure 9D- actin-MDA-MB-231

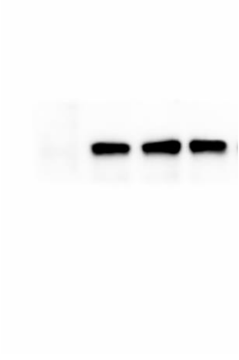

S-Figure 9D actin-HCC1937

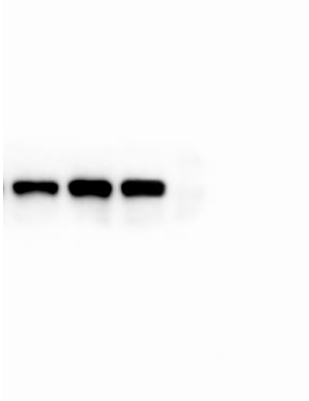

S-Figure 9D-CB1-HCC1937

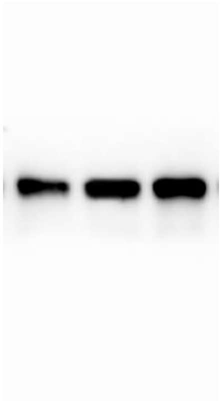

S-Figure 9D-CB1-MDA-MB-231

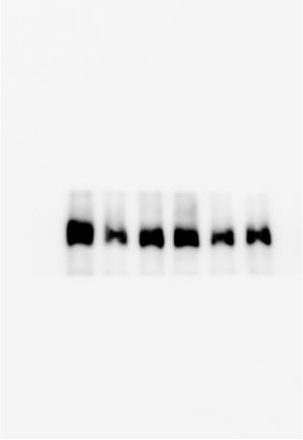

Figure-6H-SCD1

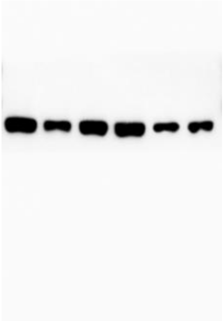

Figure-6H -FADS2

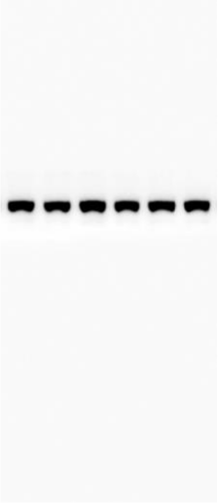

Figure-6H-β-actin

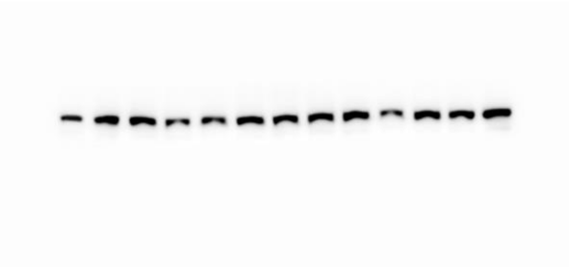

S-Figure-7E-SCD1

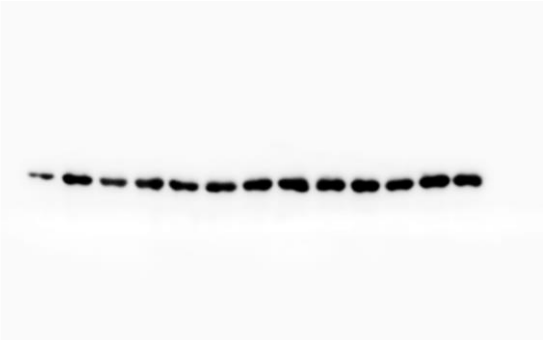

S-Figure-7E-FADS2

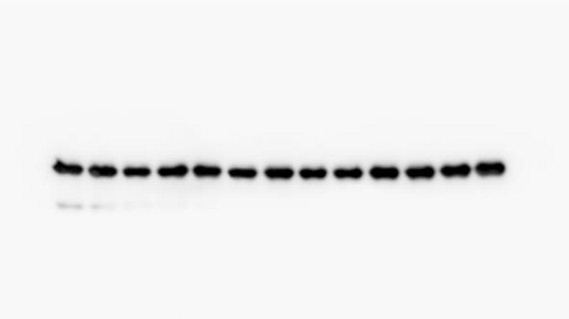

S-Figure-7E-β-actin

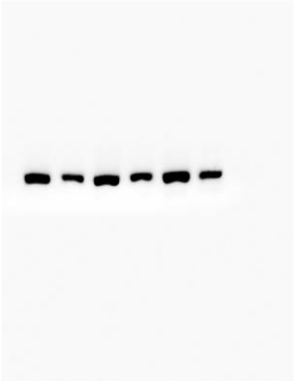

S-Figure-11G-SCD1

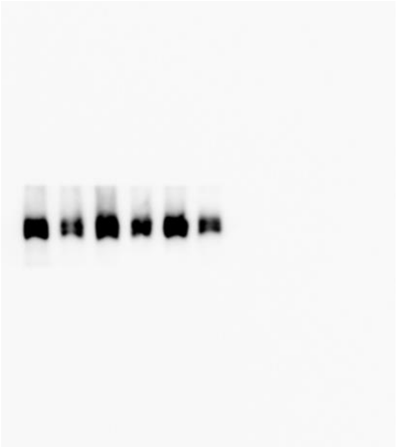

S-Figure-11G-FADS2

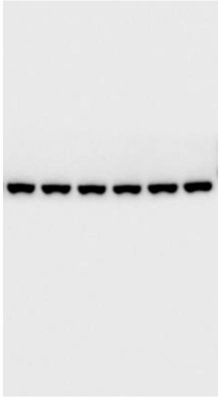

S-Figure-11G-β-actin

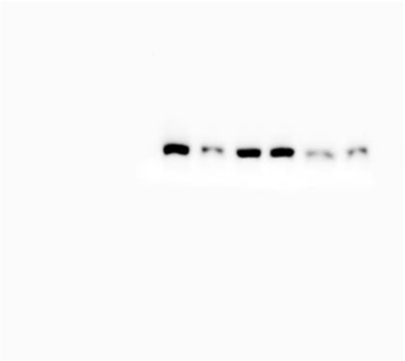

S-Figure-12F-CB1

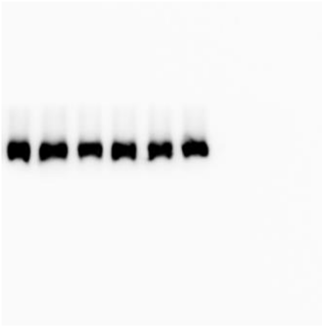

S-Figure-12F-β-actin
